# Health economic evaluations of sepsis interventions in critically ill adult patients: a systematic review

**DOI:** 10.1186/s40560-019-0412-2

**Published:** 2020-01-08

**Authors:** Alisa M. Higgins, Joanne E. Brooker, Michael Mackie, D. Jamie Cooper, Anthony H. Harris

**Affiliations:** 1 Australian and New Zealand Intensive Care Research Centre, Department of Epidemiology and Preventive Medicine, Monash University, 553 St Kilda Rd, Melbourne, Victoria 3004 Australia; 2 Department of Intensive Care and Hyperbaric Medicine, The Alfred, Melbourne, Victoria Australia; 3 Centre for Health Economics, Monash University, Melbourne, Victoria Australia

**Keywords:** Sepsis, Septic shock, Cost-effectiveness, Economic evaluation, Systematic review

## Abstract

**Background:**

Sepsis is a global health priority. Interventions to reduce the burden of sepsis need to be both effective and cost-effective. We performed a systematic review of the literature on health economic evaluations of sepsis treatments in critically ill adult patients and summarised the evidence for cost-effectiveness.

**Methods:**

We systematically searched MEDLINE, Embase, and the Cochrane Library using thesaurus (e.g. MeSH) and free-text terms related to sepsis and economic evaluations. We included all articles that reported, in any language, an economic evaluation of an intervention for the management of sepsis in critically ill adult patients. Data extracted included study details, intervention details, economic evaluation methodology, and outcomes. Included studies were appraised for reporting quality using the Consolidated Health Economic Evaluation Reporting Standards (CHEERS) checklist.

**Results:**

We identified 50 records representing 46 economic evaluations for a variety of interventions including antibiotics (*n* = 5), fluid therapy (*n* = 2), early goal-directed therapy and other resuscitation protocols (*n* = 8), immunoglobulins (*n* = 2), and interventions no longer in clinical use such as monoclonal antibodies (*n* = 7) and drotrecogin alfa (*n* = 13). Twelve (26%) evaluations were of excellent reporting quality. Incremental cost-effectiveness ratios (ICERs) ranged from dominant (lower costs and higher effectiveness) for early goal-directed therapy, albumin, and a multifaceted sepsis education program to dominated (higher costs and lower effectiveness) for polymerase chain reaction assays (LightCycler SeptiFast testing MGRADE®, SepsiTest™, and IRIDICA BAC BSI assay). ICERs varied widely across evaluations, particularly in subgroup analyses.

**Conclusions:**

There is wide variation in the cost-effectiveness of sepsis interventions. There remain important gaps in the literature, with no economic evaluations identified for several interventions routinely used in sepsis. Given the high economic and social burden of sepsis, high-quality economic evaluations are needed to increase our understanding of the cost-effectiveness of these interventions in routine clinical practice and to inform decision makers.

**Trial registration:**

PROSPERO CRD42018095980

## Background

Sepsis is recognised globally as a health priority. In 2017, the World Health Assembly and the World Health Organisation adopted a resolution to improve the prevention, diagnosis, and management of sepsis [1]. The resolution urged the United Nation Member States to implement measures to reduce both the human and health economic burden of sepsis [1]. Crude incidence estimates extrapolated from data gathered in the United States of America (USA) indicate there could be 15–19 million cases of sepsis every year worldwide [1]. Septicaemia was the most expensive condition treated in USA hospitals in 2013, with a financial burden exceeding US$23 billion [2].

It is critical to understand the cost-effectiveness of interventions designed to improve outcomes from sepsis. While there have been numerous systematic reviews summarising the evidence for effectiveness of individual interventions including early goal-directed therapy (EGDT) [3–5], fluid therapy [6–8], and corticosteroids [9–11], decision makers are also interested in which interventions deliver value for money in the context of limited health care resources. Economic evaluations assist decision making as they assess both costs and benefits, enabling a more complete consideration of the value of an intervention—what additional benefit is provided for what additional cost [12]. In 2006, Talmor and colleagues conducted a systematic review of the cost effectiveness literature in critical care medicine and found four economic evaluations of sepsis interventions, all of which were drotrecogin alpha (activated) [13]. Since that time, numerous other interventions for the treatment of sepsis have been evaluated. Wilcox and colleagues recently published a systematic review of the cost effectiveness literature in critical care medicine; however, their review was limited to intensive care interventions and English language publications [14]. Given the global burden of sepsis, we conducted a contemporary systematic review of economic evaluations of interventions for sepsis management in critically ill adult patients, including interventions delivered outside of the intensive care setting (such as in the emergency department) and articles published in languages other than English. Our objective was to summarise the evidence for the cost-effectiveness of sepsis interventions and to identify gaps in the existing literature.

## Methods

The protocol for this systematic review was registered on PROSPERO (CRD 42018095980) prior to the finalisation of the search strategies. The review was guided by the Preferred Reporting Items for Systematic Reviews and Meta-Analyses (PRISMA) statement [15].

### Search strategy

A comprehensive search of major electronic databases (Ovid MEDLINE, including Epub Ahead of Print, In-Process and Other Non-Indexed Citations; Ovid Embase Classic+Embase; and the Cochrane library, including the Health Technology Assessment database and the National Health Service Economic Evaluation Database) was conducted up to 17 July 2018 with no restrictions on the year of publication or language. Thesaurus (e.g. Medical Subject Headings; Emtree) and free-text terms relevant to sepsis and economic evaluations were used, including sepsis, septicemia, septic shock, economic evaluation, cost-benefit, cost-utility, cost-effectiveness, cost-minimisation, and critical care (full details of the search strategies are available in Additional file 1). The reference lists of included articles were also screened for any additional relevant articles.

### Study selection

All screening was performed in duplicate (AMH, JB), with discrepancies resolved by discussion with a third reviewer (MM). Screening was performed in two stages. Initially assessing titles and abstracts, we excluded articles that clearly did not meet eligibility criteria. The full text of the remaining articles was then examined. Reasons for exclusion were captured at the full article review stage.

We included all articles that reported, in any language, an economic evaluation of an intervention for the management of sepsis in critically ill adult patients. Articles published in a language other than English were translated by a medical or public health professional fluent in both English and the language of publication. An economic evaluation was defined as the comparative analysis of alternative interventions in terms of both costs (resource use) and consequences (outcomes, effects) [16]. Full economic evaluations include studies conducting cost-benefit analyses, cost-utility analyses, and cost-effectiveness analyses [12]. Cost-minimisation analyses were included where the authors pre-specified that a cost-minimisation analysis would be performed where no significant difference in outcomes was found. We included economic evaluations that were trial-based (deriving clinical and resource data from a single study) and model-based (incorporating data from various sources). Economic analyses which focused solely on costs and resources used, or which did not entail a comparator, were excluded.

Articles were also excluded for the following reasons: available in abstract form only (for example conference abstracts); reviews of existing economic evaluations that did not present new data; included patients without sepsis and results were not available separately for the sepsis subgroup; patient group was neonates or children or a mixed cohort where results were not available for the adult subgroup; or intervention was for the diagnosis of sepsis only.

### Data extraction and quality assessment

Two reviewers (AMH, JB) performed data extraction independently using data extraction forms in Covidence (Veritas Health Innovation, Melbourne, Australia). Discrepancies were resolved by discussion with a third reviewer (MM). Data extracted included study details (author, year, country, funding source), patients’ details (diagnosis, severity of illness, age), intervention details, economic evaluation methodology (time horizon, currency, discounting, perspective, type of evaluation, data sources, sensitivity analysis), and outcomes. Outcomes extracted included cost-effectiveness measures using an incremental cost-effectiveness ratio (ICER; for example cost per outcome, including cost per quality adjusted life year [QALY] and cost per life year gained) or a probability of cost-effectiveness; total costs; and health outcomes including mortality and quality of life. Interventions were reported to be dominant when they were associated with lower costs and greater effectiveness compared to the comparator, while they were reported to be dominated when associated with higher costs and lower effectiveness. Survival rates, where reported, were converted to mortality rates for consistency of outcome presentation. Corresponding authors were contacted where data required clarification. Where evaluations presented both non-discounted and discounted results, we reported the discounted results.

Quality assessment of the reporting of all included economic evaluations was performed independently by two reviewers (AMH, JB) using the 24-item Consolidated Health Economic Evaluation Reporting Standards (CHEERS) checklist [17, 18] with discrepancies resolved by discussion. A score out of 24 (or the number of applicable items) was calculated for each evaluation, with each item on the checklist assigned one point where the article adequately met the criterion. Using the method described by Hope et al. [19], a half point was awarded where the article partially filled the criterion. Where an evaluation was reported in more than one publication, both publications were used to assess reporting quality, with the evaluation being assigned the highest score from either publication for each CHEERS item. A percentage score for each evaluation was then calculated. Evaluations scoring ≥ 85% were categorised as having excellent reporting quality, 70 to < 85% as very good quality, 55 to < 70% as good quality, and evaluations scoring < 55% were classified as poor quality [19].

### Data synthesis

Studies were summarised according to the intervention evaluated. Economic evaluations of early goal-directed therapy (EGDT) for sepsis were a pre-defined subgroup, as were economic evaluations for patients meeting the criteria for septic shock. To enable comparison of results from different countries and price years, costs were converted to US dollars (USD) within the year they were performed using purchasing power parities [20] and then inflated to 2018 USD using the consumer price index [21]. A qualitative assessment of heterogeneity among trials was performed to determine whether quantitative synthesis of study results would be appropriate.

## Results

**Fig. 1:**
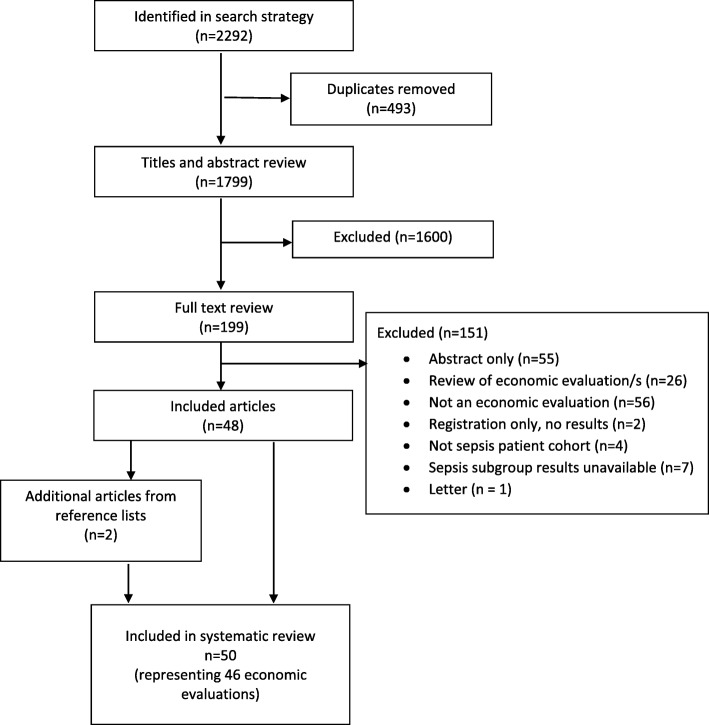
PRISMA flow diagram of study selection process

A total of 2292 records were identified from the initial search, with 1799 records remaining after removal of duplicates. Two reviewers (AMH, JB) screened titles and abstracts for all records, and a total of 199 records were retrieved for full text evaluation. An additional two records meeting the criteria were identified from reference list reviews of the included publications. A total of 50 records representing 46 health economic evaluations met criteria for inclusion (Fig. 1). Four economic evaluations had two publications each: three with health technology assessments in addition to a journal article; one with a journal article and a subsequent erratum. Records excluded following full text evaluation, along with the reason for exclusion, are listed in Additional file 2.

### Study characteristics

**Fig. 2:**
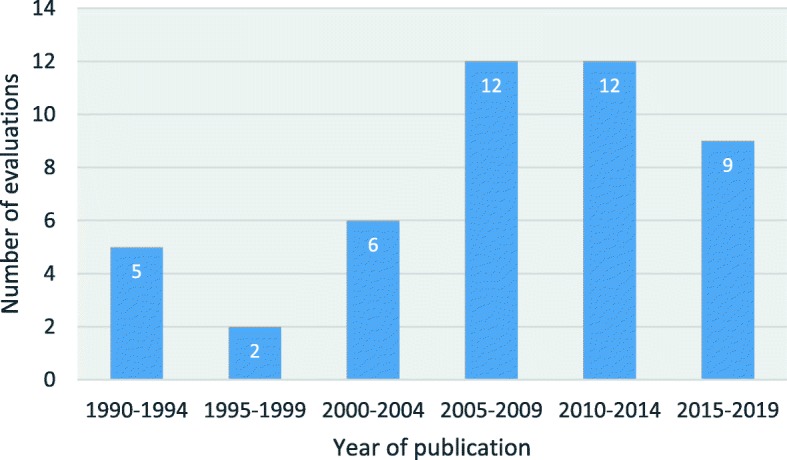
Number of published economic evaluations by 5-year period

The characteristics of included economic evaluations are summarised in Table 1 (further details are available in Additional file 3). The first evaluation was published in 1991 [64], with the majority (72%) of the evaluations published in the past 15 years (Fig. 2). All evaluations published more than 15 years ago focused on interventions that were subsequently shown to have no clinical benefit and are no longer used in the treatment of sepsis (monoclonal antibodies [HA-1A and E5] and drotrecogin alfa [activated]).

**Table 1:** General characteristics of included economic evaluations

Evaluation characteristic	Evaluations (*n* = 46), *n* (%)	Evaluation reference numbers
Interventions		
Antibiotic therapies	5 (11%)	[22–26]
Fluid therapies	2 (4%)	[27, 28]
Procalcitonin algorithms	3 (7%)	[29–31]
Immmunoglobulin therapies	2 (4%)	[32–34]
EGDT or other sepsis protocol	8 (17%)	[35–43]
Pathogen identification	4 (9%)	[44–47]
Other	2 (4%)	[48, 49]
Interventions no longer in clinical practice		
Drotrecogin alfa (activated)	13 (28%)	[50–64]
Monoclonal antibodies	7 (15%)	[65–71]
Type of evaluation		
Cost-minimisation	2 (4%)	[25, 44]
Cost-effectiveness	21 (46%)	[22, 24, 26–28, 30, 32, 39, 45, 49, 52, 54, 59, 61, 64–71]
Cost-utility	9 (20%)	[29, 31, 33, 34, 40–43, 47, 48, 63]
Cost-effectiveness and cost-utility	14 (30%)	[23, 35–38, 46, 50, 51, 53, 55–58, 60, 62]
Country		
USA	15 (33%)	[23, 28, 29, 35–37, 48, 51–53, 65, 66, 68, 70, 71]
UK	9 (20%)	[25, 31, 33, 34, 41, 42, 47, 57, 58, 62, 63, 69]
Canada	2 (4%)	[50, 59]
France	4 (9%)	[27, 45, 56, 60]
Spain	4 (9%)	[38, 44, 54, 67]
Sweden	1 (2%)	[55]
Greece	1 (2%)	[26]
Italy	1 (2%)	[24]
Netherlands	1 (2%)	[30]
Germany	2 (4%)	[32, 61, 64]
Russian Federation	1 (2%)	[22]
Brazil	2 (4%)	[39, 40]
Thailand	1 (2%)	[49]
Multinational	2 (4%)	[43, 46]
Evaluation perspective^1^		
Hospital	11 (24%)	[24, 25, 29, 30, 32, 44, 45, 49, 52, 66, 68]
Healthcare system	18 (39%)	[27, 28, 33, 34, 36, 38, 40–43, 46, 47, 50, 54, 57–62, 64, 71]
Societal	6 (13%)	[23, 35, 51, 53, 65, 70]
Not stated	11 (24%)	[22, 26, 31, 37, 39, 48, 55, 56, 63, 67, 69]
Time horizon^2^		
ICU or Hospital stay	2 (4%)	[30, 32]
28 or 30 days	3 (7%)	[45, 52, 71]
90 days	1 (2%)	[43]
6 months	1 (2%)	[31]
1 year	1 (2%)	[29]
20 years	2 (4%)	[41, 42, 59]
Lifetime	17 (37%)	[23, 24, 33–36, 38, 39, 47, 50, 51, 53, 56, 61, 63–67]
Not stated or unclear	19 (41%)	[22, 25–28, 37, 40, 44, 46, 48, 49, 54, 55, 57, 58, 60, 62, 68–70]
Funder		
Pharmaceutical company	13 (28%)	[23, 24, 27, 30, 32, 46, 48, 51, 56, 61, 62, 64, 67, 68]
Government or NFP	14 (30%)	[25, 26, 31, 33–35, 37, 38, 41–43, 45, 47, 50, 57, 58, 60]
No funding	1 (2%)	[53]
Not stated	18 (39%)	[22, 28, 29, 36, 39, 40, 44, 49, 54, 55, 59, 63, 65, 66, 69–71]
Reporting quality		
Excellent (≥ 85%)	12 (26%)	[23, 24, 30, 33–36, 38, 41, 42, 47, 51, 53, 61, 64]
Very good (70 to < 85%)	18 (39%)	[25, 28, 29, 31, 32, 37, 40, 43, 50, 52, 54, 57–60, 62, 63, 67, 68]
Good (55 to < 70%)	11 (24%)	[22, 39, 44, 45, 48, 49, 55, 56, 65, 70, 71]
Poor (< 55%)	5 (11%)	[26, 27, 46, 66, 69]

*EGDT* early goal-directed therapy, *ICU* intensive care unit, *NFP* not for profit, *UK* United Kingdom, *USA* United States of America

^1^Where studies conducted analyses from more than one perspective, the broader perspective has been reported in the table

^2^Where studies conducted more than one analysis with different time horizons, the latest time horizon has been reported in the table

Five evaluations examined antibiotic interventions [22–26], two examined fluid therapies [27, 28], eight examined EGDT or other (primarily emergency department [ED]-based) resuscitation protocols [35–42, 71], three examined procalcitonin algorithms [29–31], two examined immunoglobulin therapies [32–34], four examined methods of pathogen identification [43–46], one examined point of care lactate testing [47], and one examined immediate ICU admission [48]. The remaining 20 evaluations examined interventions no longer used in clinical practice—13 examined drotrecogin alfa (activated) (51–64), and seven examined monoclonal antibodies [64–70]. Most evaluations were cost-effectiveness and/or cost-utility analyses (44/46, 96%), with 2 (4%) cost-minimisation analyses (Table 1). Fourteen (30%) evaluations conducted both cost-effectiveness and cost-utility analyses (Table 1). Eighteen of the evaluations were trial-based evaluations, with 12 of those also using modelling to model variables not collected within the trial or extrapolate beyond the trial period. The remaining 28 evaluations used models synthesising data from various sources (Additional file 3: Table A3-1).

All evaluations were performed in patients with suspected or confirmed sepsis, with no evaluations using the updated definitions of sepsis published in 2016 [72]. The majority of evaluations were set in North America (17/46, 37%) and Europe (23/46, 50%), with only 4 (9%) from low and middle income countries or regions [22, 39, 40, 48] (Table 1).

Most evaluations were performed from the perspective of the healthcare provider (18/46, 39%) with the perspective being unclear or not stated in 24% (11/46) of evaluations (Table 1). Of the 27 evaluations which explicitly stated a time horizon, the majority used a lifetime horizon (17/27, 63%); however, 9 of these only reported costs for the initial hospitalisation. For the 19 evaluations that did not specifically state a time horizon, the majority (53%) appeared to use a lifetime horizon. Only 10/46 (22%) evaluations included costs incurred after hospital discharge.

Thirteen evaluations were funded by the manufacturer of the intervention being evaluated, while 14 were publicly funded. Eighteen evaluations did not report a funding source. The quality of reporting was comparable between those funded by the pharmaceutical industry (11/13, 85%) and those funded by government or not for profit organisations (12/13, 92%). Among evaluations funded by the pharmaceutical industry, 92% (12/13) concluded that the intervention was cost-effective compared to 64% (9/14) funded by government or not for profit organisations (see Table 1 and Additional file 3). Sensitivity analyses to determine the level of confidence associated with the economic evaluation results were conducted in 89% (41/46) of evaluations (Additional file 4).

### Reporting quality

The reporting quality varied widely, with scores ranging from 13% (3/23) [65] to 95% (21/22) [42, 50]. Twelve (26%) of the studies were found to be of excellent reporting quality, 18 (39%) of very good quality, 11 (24%) of good quality, and 5 (11%) of poor quality. The quality of evaluations published in the past 15 years was higher (average 75%) than evaluations published prior to 2005 (average 58%), with the quality of evaluations varying by intervention—88% of EGDT and resuscitation protocol evaluations were of very good or excellent quality compared to 60% of evaluations of antibiotic therapies and 25% of evaluations of pathogen identification (Additional file 4).

### Evaluation results

For evaluations of interventions currently used in clinical practice, eight (31%) studies reported incremental cost per life saved (LS) with ICERs ranging from dominant for EGDT [35, 39] to $80,852/LS (2006 €48,039/LS) for EGDT [38] (Table 2 and Additional file 3: Table A3-2). Seven studies (27%) reported incremental cost per life years gained (LYG) with ICERs ranging from dominant for albumin (over hydroxyethyl starch) [28] to $25,565/LYG (2008 $22,230/LYG) for empiric micafungin antibiotic therapy [23, 65]. Twelve (46%) evaluations reported incremental cost per QALY with ICERs ranging from dominant for a multifaceted sepsis education program to improve compliance with the Surviving Sepsis Campaign bundle [40] and a procalcitonin-guided treatment algorithm [29, 31] to dominated for PCR testing [46] (see Table 2 and Additional file 3). Only seven (27%) evaluations (of interventions in current clinical use) reported subgroup results for different illness severities with highly variable results (see Additional file 3: Table A3-3). One (4%) evaluation reported an ICER using a different measure of effectiveness (cost per antibiotic day avoided) [30], while three (12%) evaluations did not report an ICER or a probability of cost effectiveness [22, 25, 43], including the two cost-minimisation evaluations. Among currently used interventions, 77% (20/26) of evaluations concluded that the intervention was cost-effective; however, some only concluded the intervention to be cost-effectiveness under certain conditions (e.g. higher severity of illness). Due to the significant variations in methodology and reporting quality, quantitative synthesis of study results was not performed.

**Table 2:** Cost-effectiveness results for sepsis interventions

	Cost/LS (2018 USD)		Cost/LYG (2018 USD)		Cost/QALY (2018 USD)	
Intervention	*n* (%)	Range	*n* (%)	Range	*n* (%)	Range
Antibiotic therapies	1 (20%)	$70,663/LS	2 (40%)	$5,797/LYG to $25,565/LYG	1 (20%)	$39,944/QALY
Fluid therapies	1 (50%)	$8,211/LS	1 (50%)	Dominant to $839/LYG	0 (0%)	Not reported
Procalcitonin algorithms	0 (0%)	Not reported	0 (0%)	Not reported	2 (67%)	Dominant
Immunoglobulin therapies	1 (50%)	$15,738/LS	0 (0%)	Not reported	1 (50%)	$34,362/QALY
EGDT or other sepsis protocol	3 (38%)	Dominant to $80,852/LS	3 (38%)	$5,787/LYG to $14,981/LYG	5 (63%)	Dominant to $21,691/QALY
Pathogen identification	1 (25%)	$16,789/LS	0 (0%)	Not reported	2 (50%)	$2,199/QALY to Dominated
Other therapies	1 (50%)	$4,029/LS	0 (0%)	Not reported	1 (50%)	$34,984/QALY
Interventions no longer in clinical practice						
Drotecogin alfa (activated)	3 (23%)	$79,418/LS to $233,600/LS	10 (77%)	$2,696/LYG to $48,618/LYG	9 (69%)	$3,901/QALY to $71,248/QALY
Monoclonal antibodies	4 (57%)	$24,719/LS to $379,579/LS	3 (43%)	$2,679/LYG to $1,830,283/LYG	0 (0%)	Not reported

*EGDT* early goal directed therapy, *LS* life saved, *LYG* life years gained, *QALY* quality-adjusted life year, *USD* United States dollar

In evaluations modelling life expectancy following sepsis (*n* = 32), most (12/32, 38%) used a single adjustment factor of 0.51 [73] to reduce age and gender-specific population life expectancy to account for the higher long-term mortality risk following sepsis [23, 35–38, 40, 50, 53–55, 59, 60]. Among the 23 cost-utility evaluations, the majority (61%) used a single utility value multiplied by life expectancy to determine QALYs, and only one evaluation prospectively measured quality of life [42]. Most cost-utility evaluations varied utility weights in sensitivity analyses, with the majority finding that the utility weight (across the range varied) did not impact on conclusions about cost-effectiveness.

## Discussion

We conducted a comprehensive systematic review of economic evaluations of sepsis interventions and identified 50 publications representing 46 economic evaluations. The evaluations were of a variety of interventions, some of which have since been shown to have no clinical benefit and are no longer in use (e.g. drotrecogin alfa [activated] and monoclonal antibodies). There was significant heterogeneity in design of the evaluations, including in the outcome measures used, the range of costs included, the time horizon, and the evaluation perspective. This prevented a quantitative synthesis of results, and interpretation of any such results would be unclear. The narrative synthesis of results indicated wide variation in ICERs.

Economic evaluations are constrained by limited availability of high quality evidence from randomised controlled trials (RCTs) and limited data on long term outcomes including quality of life. For health care decision makers to be able to make accurate decisions about the economic effects of sepsis interventions, sufficient data needs to be available. Existing studies of the long-term natural history of sepsis have typically had small sample sizes or low follow-up rates. Better characterisation of recovery following sepsis can reduce the need to make numerous assumptions about the trajectory of outcomes in economic evaluations of interventions in sepsis. Whilst clinical trials can provide effectiveness data with high internal validity, they are often limited in the range of resource use and outcome data collected, or the length of follow-up. As a result, most economic evaluations need to model resource use and/or longer-term outcomes (such as life expectancy) to determine the cost-effectiveness of the intervention. Only five of the evaluations included in our review did not have any modelling component. Sepsis is known to have long term consequences including a higher risk of readmissions, cardiovascular disease, cognitive impairment, and death [74]. Only 17 of the evaluations included in the review reported using a lifetime horizon (with a further 10 of 19 that did not state a time horizon appearing to use a lifetime horizon). However, despite evidence showing that 40% of sepsis patients will be readmitted to hospital within 90 days of discharge [75], 19 (70%) of the evaluations with a stated or assumed lifetime horizon did not include any costs beyond the initial hospitalisation. In interventions that improve hospital survival, this may result in an overestimate of cost-effectiveness as the long-term healthcare costs of survivors are not included.

Many of the evaluations included in our review used data from non-randomised trials or from single-centre RCTs. For example, 6 of the 8 evaluations of EGDT and other sepsis protocols used data from a single-centre trial or from non-randomised pre-post studies. The reliance on economic evaluations using such data can result in an overrepresentation of the cost-effectiveness of an intervention. All pre-post studies and the single-centre EGDT RCT by Rivers and colleagues [76] showed a significant survival benefit at hospital discharge associated with sepsis protocols. The majority of economic evaluations assumed that the survival benefit was maintained over time. Three subsequent multi-centre RCTs of EGDT showed no survival benefit [41, 77, 78], with subsequent removal of the recommendation for EGDT from the Surviving Sepsis Campaign guidelines [79]. All economic evaluations conducted prior to publication of the three multicentre RCTs concluded that EGDT and other sepsis protocols were cost-effective, with the two evaluations conducted following the multi-centre RCTs both concluding that EGDT was not cost-effective [41, 42, 71]. This example shows the problems that can arise when drawing conclusions about cost effectiveness from single-centre or non-randomised trials or where sufficient outcome data is unavailable.

Given the high morbidity and mortality from sepsis, the high economic burden, and the large numbers of clinical trials performed, relatively few economic evaluations have been performed of sepsis interventions. The Surviving Sepsis Campaign published evidence based guidelines for the treatment of sepsis with the aim of reducing mortality and morbidity [79]. In our review, we identified no economic evaluations for several of the interventions mentioned in the guidelines. The SSC guidelines recommend resuscitation with crystalloid fluid (with the addition of albumin when patients require substantial amounts of crystalloids); however, we identified only two economic evaluations of fluid therapy [27, 28], neither of which incorporated a measure of cost/QALY and one of which was poor quality [27]. The use of corticosteroids in sepsis has been a topic of immense interest, with numerous systematic reviews conducted [9–11]. One recent systematic review identified 42 RCTs of corticosteroids in sepsis [11], yet our review identified no economic evaluations of corticosteroids. The SSC guidelines recommend norepinephrine as the first choice vasopressor, and despite a recent systematic review identifying 32 RCTs of vasopressors for the treatment of septic shock [80], we found no economic evaluations for this intervention. These examples show the large gaps that exist in the literature for the cost-effectiveness of many sepsis interventions. It is essential that the value for money of many routinely used sepsis interventions is determined.

Wilcox and colleagues recently summarised economic evaluations of interventions in critical care and despite searching the literature from 1993 to 2018, identified just 20 evaluations of sepsis interventions, compared to 46 evaluations of sepsis interventions in the current review [14]. Our review differs from that of Wilcox and colleagues in that we did not restrict our search by year of publication, language, or location of critically ill patients (Wilcox and colleagues limited their review to the ICU setting). Only two of the evaluations in the current review were published prior to 1993, and three were published in a language other than English and therefore not eligible in the review by Wilcox and colleagues. The additional evaluations identified in the current review were for a variety of interventions, including both evaluations of immunoglobulin therapies, and all five evaluations of antibiotic therapies (four of which were published in English).

A key strength of our review is that a comprehensive search strategy was developed, encompassing multiple electronic databases, increasing the likelihood of identifying all economic evaluations of sepsis interventions. Further, the current review did not limit the search by date or language of publication. Study selection, data extraction, and reporting quality assessment were independently undertaken by two reviewers. However, reviewers were not blinded to the authors or journal of publication which may have influenced results, particularly when completing the reporting quality checklist. The quality assessment tool used in the review, the CHEERS checklist, indicated that the reporting quality of 89% of the included economic evaluations was good to excellent. However, while the CHEERS checklist assesses quality of the reporting of various aspects of an economic evaluation, it does not assess the suitability of the methodology reported, nor the quality of the data that was used to inform the economic evaluation.

Our review did not identify any economic evaluations of interventions using the current sepsis-3 definitions (sepsis-3) [72]. As an inclusion criterion of the current review was that the economic evaluation was in critically ill septic patients, it is likely that patients in most of the evaluations would meet current sepsis definitions. It is not possible, however, to determine the impact of the new definitions on the economic evaluation results given the data available. Future economic evaluations should ensure that the included population meets current sepsis definitions.

## Conclusions

This systematic review identified considerable heterogeneity in the design of economic evaluations for interventions in sepsis and wide variation in reported results. It also identified important gaps in the literature, with no economic evaluations identified for several interventions routinely used in sepsis. Given the high economic and social burden of sepsis, high quality economic evaluations are needed to increase our understanding of the cost-effectiveness of these interventions in routine clinical practice and to inform decision makers.

## Supplementary information

**Additional file 1.** MEDLINE, Embase and Cochrane library search strategies

**Additional file 2.** Publications excluded at full text stage

**Additional file 3.** Tables A3-1 to A3-3 Characteristics and results of included evaluations

**Additional file 4.** CHEERS checklist and assessment of included economic evaluations

## Data Availability

Available from the corresponding author on reasonable request.

## Abbreviations

CHEERS: Consolidated Health Economic Evaluation Reporting Standards
EGDT: Early goal-directed therapy
ICER: Incremental cost-effectiveness ratio
ICU: Intensive care unit
LS: Lives saved
LYG: Life years gained
PCR: Polymerase chain reaction
PRISMA: Preferred Reporting Items for Systematic Reviews and Meta-Analyses
QALY: Quality-adjusted life year
USA: United States of America
USD: US dollars
