# Supplementary material for: Health economic evaluations of sepsis interventions in critically ill adult patients: a systematic review
Source: J Intensive Care. 2020 Jan 8;8:5. doi: 10.1186/s40560-019-0412-2 (PMC6950865; doi:10.1186/s40560-019-0412-2)
Supplement: Supplementary file 1 — Additional file 1. MEDLINE, Embase and Cochrane library search strategies [file 40560_2019_412_MOESM1_ESM.docx]

**Additional file 1. MEDLINE, Embase and Cochrane library search strategies**

**Additional file for:**

Higgins AM, Brooker J, Mackie M, Cooper DJ and Harris A

Health economic evaluations of sepsis interventions in critically ill adult patients: a systematic review

**Ovid MEDLINE(R) Epub Ahead of Print, In-Process & Other Non-Indexed Citations, Ovid MEDLINE(R) Daily, Ovid MEDLINE and Versions(R)** 1946 to July 13, 2018

| 1. exp "Costs and Cost Analysis"/ |
| --- |
| 2. exp economics, hospital/ or exp economics, medical/ or exp economics, nursing/ or exp economics, pharmaceutical/ |
| 3. ((cost* adj2 (benefit* or effect* or minimi* or utilit* or outcome*)) or (economic* adj evaluat*)).mp. |
| 4. or/1-3 |
| 5. exp Sepsis/ |
| 6. (sepsis or septi*).mp. |
| 7. 5 or 6 |
| 8. exp Critical care/ or exp Intensive Care Units/ or Critical illness/ |
| 9. (((critical or intensive) adj2 (treatment* or care)) or (critical* adj2 (ill* or patient*)) or ICU or ITU or CCU).mp. |
| 10. Emergency Medicine/ or exp Emergency Treatment/ or Evidence-Based Emergency Medicine/ or exp Emergency Medical Services/ or Emergencies/ |
| 11. ((emergency or emergencies) adj3 (department* or room* or ward* or treatment* or medicine* or service* or accident*)).mp. |
| 12. 7 and (8 or 9 or 10 or 11) |
| 13. (septic shock or severe sepsis).mp. |
| 14. 12 or 13 |
| 15. Intensive Care, Neonatal/ or exp Intensive Care Units, Pediatric/ or Neonatal sepsis/ |
| 16. (14 not 15) and 4 |
| 17. limit 16 to animals |
| 18. 16 not 17 |

**Ovid Embase Classic+Embase** 1947 to 2018 July 13

| 1. exp Economic Evaluation/ |
| --- |
| 2. ((cost* adj2 (benefit* or effect* or minimi* or utilit* or outcome*)) or (economic* adj evaluat*)).mp. |
| 3. or/1-2 |
| 4. exp Sepsis/ |
| 5. (sepsis or septi*).mp. |
| 6. 4 or 5 |
| 7. exp Intensive care/ or Critically ill patient/ |
| 8. (((critical or intensive) adj2 (treatment* or care)) or (critical* adj2 (ill* or patient*)) or ICU or ITU or CCU).mp. |
| 9. exp Emergency Treatment/ or Emergency Ward/ or Emergency health service/ or Hospital emergency service/ or Emergency medicine/ or Emergency patient/ |
| 10. ((emergency or emergencies) adj3 (department or room or ward or treatment or medicine or service or accident)).mp. |
| 11. 6 and (7 or 8 or 9 or 10) |
| 12. (septic shock or severe sepsis).mp. |
| 13. Newborn intensive care/ or Newborn intensive care nursing/ or Pediatric intensive care nursing/ or Neonatal intensive care unit/ or Pediatric intensive care unit/ or exp Fetus monitoring/ or Newborn monitoring/ or Pediatric advanced life support/ or Neonatal sepsis/ or Pediatric emergency medicine/ or Pediatric advanced life support/ |
| 14. (11 or 12) not 13 |
| 15. 3 and 14 |
| 16. limit 15 to animal studies |
| 17. 15 not 16 |

**Cochrane Library**, inception to 17 July 2018

#1 MeSH descriptor: [Costs and Cost Analysis] explode all trees

#2 MeSH descriptor: [Economics, Hospital] explode all trees

#3 MeSH descriptor: [Economics, Medical] explode all trees

#4 MeSH descriptor: [Economics, Nursing] explode all trees

#5 MeSH descriptor: [Economics, Pharmaceutical] explode all trees

#6 ((cost* near/2 (benefit* or effect* or minimi* or utilit* or outcome*)) or (economic* near/2 evaluat*)):ti,ab,kw

#7 #1 or #2 or #3 or #4 or #5 or #6

#8 MeSH descriptor: [Sepsis] explode all trees

#9 (sepsis or septi*):ti,ab,kw

#10 MeSH descriptor: [Critical Care] explode all trees

#11 MeSH descriptor: [Critical Illness] explode all trees

#12 MeSH descriptor: [Intensive Care Units] explode all trees

#13 (((critical or intensive) near/2 (treatment* or care)) or (critical* near/2 (ill* or patient*)) or ICU or ITU or CCU):ti,ab,kw

#14 MeSH descriptor: [Emergency Medicine] explode all trees

#15 MeSH descriptor: [Emergency Treatment] explode all trees

#16 MeSH descriptor: [Evidence-Based Emergency Medicine] explode all trees

#17 MeSH descriptor: [Emergency Medical Services] explode all trees

#18 MeSH descriptor: [Emergencies] explode all trees

#19 ((emergency or emergencies) near/3 (department* or room* or ward* or treatment* or medicine* or service* or accident*)):ti,ab,kw

#20 (#8 or #9) and (#10 or #11 or #12 or #13 or #14 or #15 or #16 or #17 or #18)

#21 ("septic shock" or "severe sepsis"):ti,ab,kw

#22 MeSH descriptor: [Intensive Care, Neonatal] explode all trees

#23 MeSH descriptor: [Intensive Care Units, Pediatric] explode all trees

#24 MeSH descriptor: [Neonatal Sepsis] explode all trees

#25 (#20 or #21) not (#22 or #23 or #24)

#26 #7 and #25
