# Supplementary material for: Health economic evaluations of sepsis interventions in critically ill adult patients: a systematic review
Source: J Intensive Care. 2020 Jan 8;8:5. doi: 10.1186/s40560-019-0412-2 (PMC6950865; doi:10.1186/s40560-019-0412-2)
Supplement: Supplementary file 2 — Additional file 2. Publications excluded at full text stage [file 40560_2019_412_MOESM2_ESM.docx]

**Additional file 2. Publications excluded at full text stage**

**Additional file for:**

Higgins AM, Brooker J, Mackie M, Cooper DJ and Harris A

Health economic evaluations of sepsis interventions in critically ill adult patients: a systematic review

| **Exclusions reason: Abstract only** |
| --- |
| 1. Ahmed A, Adamo M, Balchandra S. The real costs of treating early post-operative leaks following sleeve gastrectomy procedures. Obesity Surgery. 2015;1:S45. |
| 2. Allen TL, Jarman AF, Dong L, Merrill A, Nelson N, Miller IRR, et al. Quality lowers cost: The cost effectiveness of a multicenter treatment bundle for severe sepsis and septic shock. Academic Emergency Medicine. 2015;1:S64. |
| 3. Amalakuhan B, Srouji N, Parvathaneni A, Deveney R, Rich J, Farzin S. A hospital wide "sepsis-alert" system: Going a step beyond classical "early goal directed therapy" for severe sepsis/septic shock and improving mortality and health-care expenditure on a global level. American Journal of Respiratory and Critical Care Medicine Conference: American Thoracic Society International Conference, ATS. 2012;185(Meeting Abstracts). |
| 4. Auzinger G, Playford G, Graham C, Narula H, Charbonneau C, Weinstein D, et al. Cost-effectiveness analysis of anidulafungin in the treatment of candidaemia. Critical Care. 2013;2:S32. |
| 5. Berto P, Antonelli M, Ronco C, Cruz D, Melotti RM. Cost-effectiveness of polymyxin B immobilized fiber column and conventional medical therapy in the management of severe abdominal sepsis in Italy. Value in Health. 2010;13(3):A5. |
| 6. Donaldson C, Rech M, Sterk E. Impact of IV push antibiotics on time to empiric antibiotic administration in septic patients. Critical Care Medicine. 2018;46(Supplement 1):739. |
| 7. Dravid T, Pawar B, Bhurke B, Akole P, Rajhans P, Jog S. Activated protein C: Cost matters! Critical Care. 2010;1:S136. |
| 8. Fernando S, Reardon P, Van Katwyk S, Thavorn K, Tanuseputro P, Rosenberg E, et al. Outcomes and costs of patients with sepsis transferred to a tertiary care intensive care unit. Critical Care Medicine. 2018;46(Supplement 1):691. |
| 9. Fernando SM, Rochwerg B, Reardon PM, Thavorn K, Shapiro NI, Seely AJE, et al. Increased mortality and costs in emergency department sepsis patients with delayed intensive care unit admission. Canadian Journal of Emergency Medicine. 2018;20(Supplement 1):S24. |
| 10. Gardner-Gray J, Jaehne A, McGregor K, Clark A, Brown S, Schlichting A, et al. Cost savings associated with compliance to an early sepsis intervention strategy. Chest Conference. 2012;142(4 Suppl 1). |
| 11. Gotur D, Masud F, Halfon R, Birney P. Quality initiatives to improve sepsis mortality in a tertiary hospital: An 8-year outcomes measure. Critical Care Medicine. 2018;46(Supplement 1):572. |
| 12. Heimann S, Cornely OA, Wisplinghoff H, Vehreschild M, Franke B, Glossmann J, et al. Cost analysis of candidaemia in patients on the intensive care unit. Clinical Microbiology and Infection. 2012;3:180. |
| 13. Heimann S, Cornely OA, Wisplinghoff H, Vehreschild MJ, Franke B, Glossmann J, et al. Cost-of-illness analysis of candidemia in patients on the intensive care unit. Value in Health. 2012;15(7):A390. |
| 14. Heimann S, Cornely OA, Wisplinghoff H, Vehreschild MJGT, Franke B, Glossmann J, et al. Cost-of-illness analysis of Candidemia in patients on the intensive care unit. Mycoses. 2012;55:325-6. |
| 15. Latham H, Bengtson C, Satterwhite L, Stites M, Sahatjian J, Hansell D, et al. Sepsis resuscitation based on stroke volume optimization improves outcome and reduces cost of care. Critical Care Medicine. 2018;46:710. |
| 16. Popescu C, Popescu GA, Lobodan A, Dulama R, Niculescu D, Tenase D, et al. In the era of broad spectrum antibiotics, is ampicillin still an option? BMC Infectious Diseases Conference. 2013;13(Supplement 1). |
| 17. Sutherland T, Foreman L, Neumann P, Talmor D. Cost-effectiveness research in critical care between 2004-2013: A systematic review. Critical Care Medicine. 2015;1:230. |
| 18. Westwood DA. Utility of blood cultures from patients discharged from the adult emergency department. European Journal of Emergency Medicine. 2007;14(5):283. |
| 19. Zaitsev A, Tyrsin O, Morozov A. Clinico-economic evaluation of treatment of community-acquired pneumonia (CAP) complicated by sepsis with moxifloxacin compared to ceftriaxone-azithromycin. Value in Health. 2011;14:A269. |
| 20. Farrugia A, Balboni A, Cassar J, Kimber MC. Colloid treatment in sepsis patients in intensive care-use of albumin vs hydroxyethyl starch (HES) is cost-effective in a decision analysis model. Vox Sanguinis. 2012;1:238. |
| 21. Ranzani OT, Noritomi DT, Barbosa M, Machado FR, Ferreira EM, Paula IC, et al. Implementation of surviving sepsis campaign in a group of private hospitals in brazil: Effects on outcomes, costeffectiveness and temporal trends in a propensity analysis. Intensive Care Medicine. 2012;1:S279. |
| 22. Barlozek M, Nowak A, Ruebesam T, Gockel U. Number needed to treat and cost per life saved of adjunctive igmenriched immunoglobulin treatment of sepsis in Germany. Infection. 2017;45(Supplement 1):S45. |
| 23. Edamoto Y, Suda R, Kumazawa K, Hashimoto M, Saito Y, Shimizu T. Direct hemoperfusion with polymyxin B-immobilized fiber column (PMX-DHP) can improve the prognosis and medical expense. Inflammation Research. 2010;1:S135. |
| 24. Boldger A, Davies J, Johnson A, Wheeler M. Procalcitonin in an intensive treatment unit (ITU): An economic evaluation. Clinical Chemistry and Laboratory Medicine. 2014;52(11):eA361. |
| 25. Farrugia A, Bansal M. Choice of colloid fluid therapy in septic patients: A comparative cost-effectiveness analysis of crystalloid albumin and hydroxyethyl starch. Intensive Care Medicine. 2013;2:S297. |
| 26. Garces K. Activated protein C for severe sepsis2002; (4):[4 p.]. Available from: http://cochranelibrary-wiley.com/o/cochrane/clhta/articles/HTA-32002000369/frame.html. |
| 27. Grau S, Pozo J, Roma E, Salavert M, Barrueta J, Peral C, et al. Pharmacoeconomic analysis of anidulafungin, micafungin, caspofungin and fluconazole in the treatment of candidemia and/or invasive candidiasis in non-neutropenic adult patients in Spain. Value in Health. 2015;18:A584-A5. |
| 28. Jog S, Dravid T, Phadke P, Kulkarni R, Bapat N, Akole P, et al. Drotrecogin alpha-cutting cost, not corners! Intensive Care Medicine. 2009;1:S171. |
| 29. Jones AE, Troyer J. Cost-Effectiveness of an emergency department based early sepsis resuscitation protocol: A prospective study. Academic Emergency Medicine. 2010;1:S5. |
| 30. Kron J, Kron S, Wenkel R, Schuhmacher HU, Thieme U, Leimbach T, et al. Extended daily on-line high volume haemodiafiltration in septic multiple organ failure: Outcome and costs. NDT Plus. 2010;3:iii366. |
| 31. Krysanov I, Krysanova V. Cost-effectiveness analysis of the application of ertapenem for the treatment of community-acquired complicated intra-abdominal infections. Value in Health. 2014;17(7):A678. |
| 32. Kumar A. Use of molecular diagnostic technique using 'multiplex nuclic acid amplification' in the management of sepsis patients at a tertiary care medical oncology intensive care unit. Indian Journal of Critical Care Medicine. 2013;2:9-10. |
| 33. Lai PS, Shrime MG, Ferket BS, Scott DJ, Lee J, Celi LA, et al. Using Markov models to determine the optimal duration for a trial of intensive care in patients with active cancer and septic shock. American Journal of Respiratory and Critical Care Medicine. 2012;185(Meeting Abstracts: Conference: American Thoracic Society International Conference, ATS). |
| 34. Leisman D, Bianculli A, Doerfler M, Gribben J, Andersen R, D'Angelo J, et al. Survival benefit and cost savings from emergency department compliance with a basic 3-hour sepsis bundle in a multisite, prospective, observational study. Academic Emergency Medicine. 2016;1:S18. |
| 35. Leisman D, Wie B, Tevere J, Brody D, D'Angelo J, Ward MF, et al. Can emergency department compliance with a basic 3-hour sepsis bundle reduce mortality, ICU utilization, length of stay, and hospital costs without reliance on physiologic endpoints? Academic Emergency Medicine. 2015;1:S79. |
| 1. Molina FJ, Cortes JA, Caceres HA, Soto R, Lemos Luengas EV. Cost-effectiveness analysis of the therapy for the invasive Candidiasis in Colombia. International Journal of Infectious Diseases. 2010;1:e123. |
| 37. Molina FJ, Cortes JA, Soto R, Lemos EV, Gutierrez-Ardila MV, Nunez SM. Cost-effectiveness analysis of antifungal treatments available in Colombia for the treatment of invasive candidiasis. Value in Health. 2012;15(4):A242. |
| 38. Navas A, Suarez D, Goma G, Gili G, Ferrer R, Artigas A. Cost of managing severe sepsis patients treated with toraymyxin in spain. Intensive Care Medicine. 2013;2:S479-S80. |
| 39. Nowak A, Barlozek M, Ruebesam T, Gockel U. Cost per life saved of adjunctive igm-enriched immunoglobulin treatment of sepsis in Germany. Value in Health. 2017;20(9):A784. |
| 40. Peric A, Kovaeevic SV, Surbatovic M, Antunovic M. Economic aspects of the use of carbapenems in critically ill patients. European Journal of Hospital Pharmacy. 2017;24(Supplement 1):A23. |
| 41. Reboli A, Rotstein C, Kett D, Shorr A, Chambers R, Hux M, et al. Economic evaluation of anidulafungin (Eraxis) versus intravenous fluconazole in the treatment of hospital inpatients diagnosed with candidaemia and other forms of invasive candidiasis. Clinical Microbiology and Infection. 2009;15(S4):S502. |
| 42. Singer AJ, Chase K, Murphy P, Thode Jr HC. The financial effect of point-of-care lactates in ED patients with sepsis. Academic Emergency Medicine. 2015;1:S385. |
| 43. Soares MO. The value of information of a multicentre randomised controlled trial of intravenous immunoglobulin for sepsis (severe sepsis and septic shock). Value in Health [Internet]. 2011; 14(7):[A240 p.]. Available from: http://ovidsp.ovid.com/ovidweb.cgi?T=JS&CSC=Y&NEWS=N&PAGE=fulltext&D=emed13&AN=70574289. |
| 44. Suarez D, Ferrer R, Artigas A, Garnacho-Montero J, Levy MM. Cost-effectiveness of the surviving sepsis campaign protocol for severe sepsis in Spain. Value in Health. 2009;12(7):A427-A8. |
| 45. Van Der Maas M, Kip M, Steuten L. Estimating the additional indirect cost savings of a procalcitonalgorithm in adult ICU patients with sepsis, as achieved through reduction in antibiotic resistance and C. Difficile infections. Value in Health. 2015;18(7):A352-A3. |
| 46. Walke T, Chalfin D, Lee J, Rivers E. Cost-effectiveness of a rapid and accurate test for diagnosing infection in severe sepsis and septic shock patients. Critical Care. 2010;1:S18. |
| 47. Ward MJ, Self WH, Singer AJ, Lazar D, Pines JM. Cost-effectiveness analysis of early point-of-care lactate testing in the emergency department. Academic Emergency Medicine. 2016;1:S26-S7. |
| 48. Weiss M, Lautenschlager F, Porzsolt F. SSC guideline limit in surgical patients with septic shock. Infection. 2011;2:S137-S8. |
| 49. Wilke M. From theory to practice: Saving costs by adding biomarkers to infection management. Biochimica Clinica. 2013;1:S76-S7. |
| 50. Carrington J, Barnes J. Making ICU admissions smarter, not making them harder: Lowering ICU admission thresholds improves outcomes and resource utilization. Chest Conference. 2015;148(4 Meeting Abstract). |
| 51. Ferrer R. Cost-effectiveness of the surviving sepsis campaign in Spain. Intensive Care Medicine. 2009;1:S12. |
| 52. Guidet B. [Medical-economic evaluation of severe sepsis]. Annales Francaises d Anesthesie et de Reanimation. 2003;22 Spec No 1:28-30. |
| 53. Huntley M, Schutzer-Weissmann J, Knight-George A. The use of procalcitonin in sepsis: A district general intensive care unit experience. Anaesthesia. 2011;66(1):68-9. |
| 54. Lee TJ, Lee H, Park B. Clinical effectiveness and cost-effectiveness of a Polymyxin B-immobilized hemoperfusion cartage for the treatment of severe sepsis: A systemic review and economic evaluation. Value in Health. 2009;12(3):A113. |
| 55. Hohn A, Schroeder S. Effects of a procalcitonin-guided protocol on length of antibiotic therapy in patients with severe sepsis and septic shock. Infection. 2013;1(Supplement):S34. |
| **Exclusions reason: Clinical trial registration – results not available** |
| 56. Nct. Procalcitonin-guided Treatment on Duration of Antibiotic Therapy and Cost in Septic Patients2014. Available from: http://cochranelibrary-wiley.com/o/cochrane/clcentral/articles/606/CN-01547606/frame.html. |
| 57. Nct. Clinical and Economic Outcome of Simvastatin in Critically Ill Septic Patient2014. Available from: http://cochranelibrary-wiley.com/o/cochrane/clcentral/articles/830/CN-01543830/frame.html. |
| **Exclusion reason: Sepsis subgroup cost-effectiveness results not available** |
| 58. Davey PG, Parker SE, Orange G, Malek M, Dodd T. Prospective audit of costs and outcome of aminoglycoside treatment and of therapy for gram-negative bacteraemia. Journal of Antimicrobial Chemotherapy. 1995;36(3):561-75. |
| 59. Edwards SJ, Campbell HE, Plumb JM. Cost-utility analysis comparing meropenem with imipenem plus cilastatin in the treatment of severe infections in intensive care. European Journal of Health Economics. 2006;7(1):72-8. |
| 60. Hamandi B, Husain S, Humar A, Papadimitropoulos EA. Impact of infectious disease consultation on the clinical and economic outcomes of solid organ transplant recipients admitted for infectious complications. Clinical Infectious Diseases. 2014;59(8):1074-82. |
| 61. Harwan WA, Abbassi MM, El-Attar MM, Farid SF. Pharmacoeconomic study of antibiotics used in the treatment of lower respiratory tract infections in ICU patients: A case study in an Egyptian hospital. Bulletin of Faculty of Pharmacy. 2014;52(1):135-44. |
| 62. Heyland DK, Johnson AP, Reynolds SC, Muscedere J. Procalcitonin for reduced antibiotic exposure in the critical care setting: a systematic review and an economic evaluation. Critical Care Medicine. 2011;39(7):1792-9. |
| 63. Ruiz-Ramos J, Frasquet J, Roma E, Poveda-Andres JL, Salavert-Leti M, Castellanos A, et al. Cost-effectiveness analysis of implementing an antimicrobial stewardship program in critical care units. Journal of Medical Economics. 2017;20(6):652-9. |
| 64. Grau S, Pozo JC, Roma E, Salavert M, Barrueta JA, Peral C, et al. Cost-effectiveness of three echinocandins and fluconazole in the treatment of candidemia and/or invasive candidiasis in nonneutropenic adult patients. ClinicoEconomics and Outcomes Research. 2015;7:527-35. |
| **Exclusion reason: Duplicate** |
| 65. Angus DC, Linde-Zwirble WT, Clermont G, Ball DE, Basson BR, Ely EW, et al. Cost-effectiveness of drotrecogin alfa (activated) in the treatment of severe sepsis. Critical Care Medicine. 2003;31(1):1-11. |
| 66. Assuncao MS, Teich V, Shiramizo SC, Araujo DV, Carrera RM, Serpa Neto A, et al. The cost-effectiveness ratio of a managed protocol for severe sepsis (Provisional abstract)2014; 29(4):[692.e1-e6 pp.]. Available from: http://cochranelibrary-wiley.com/o/cochrane/cleed/articles/NHSEED-22014023163/frame.html. |
| 67. Berto P, Ronco C, Cruz D, Melotti RM, Antonelli M. Cost-effectiveness analysis of polymyxin-B immobilized fiber column and conventional medical therapy in the management of abdominal septic shock in Italy (Provisional abstract) 2011; 32(4):[331-40 pp.]. Available from: http://cochranelibrary-wiley.com/o/cochrane/cleed/articles/NHSEED-22012000017/frame.html. |
| 68. Champunot R, Thawitsri T, Kamsawang N, Sirichote V, Nopmaneejumruslers C. Cost effectiveness analysis of an initial ICU admission as compared to a delayed ICU admission in patients with severe sepsis or in septic shock (Provisional abstract) 2014; 97(Supplement 1):[S102-S7 pp.]. Available from: http://cochranelibrary-wiley.com/o/cochrane/cleed/articles/NHSEED-22014023165/frame.html. |
| 69. Costa V, Brophy JM. Drotrecogin alfa (activated) in severe sepsis: a systematic review and new cost-effectiveness analysis (Provisional abstract) 2007; 7(5):[1-11 pp.]. Available from: http://cochranelibrary-wiley.com/o/cochrane/cleed/articles/NHSEED-22007001809/frame.html. |
| 70. Davies A, Ridley S, Hutton J, Chinn C, Barber B, Angus DC. Cost effectiveness of drotrecogin alfa (activated) for the treatment of severe sepsis in the United Kingdom (Structured abstract)2005; 60(2):[155-62 pp.]. Available from: http://cochranelibrary-wiley.com/o/cochrane/cleed/articles/NHSEED-22005000288/frame.html. |
| 71. Fowler RA, Hill-Popper M, Stasinos J, Petrou C, Sanders GD, Garber AM. Cost-effectiveness of recombinant human activated protein C and the influence of severity of illness in the treatment of patients with severe sepsis2003; 18(3):[181-91 pp.]. Available from: http://cochranelibrary-wiley.com/o/cochrane/cleed/articles/NHSEED-22003001497/frame.html. |
| 72. Franca LR, Launois R, Lay K, Aegerter P, Bouhassira M, Meshaka P, et al. Cost-effectiveness of drotrecogin alfa (activated) in the treatment of severe sepsis with multiple organ failure2006; 22(1):[101-8 pp.]. Available from: http://cochranelibrary-wiley.com/o/cochrane/cleed/articles/NHSEED-22006008089/frame.html. |
| 73. Green C, Dinnes J, Takeda A, Shepherd J, Hartwell D, Cave C, et al. Clinical effectiveness and cost-effectiveness of drotrecogin alfa (activated) (Xigris (R)) for the treatment of severe sepsis in adults: a systematic review and economic evaluation2005; (4):[1 p.]. Available from: http://cochranelibrary-wiley.com/o/cochrane/clhta/articles/HTA-32005000185/frame.html. |
| 74. Green C, Dinnes J, Takeda A, Shepherd J, Hartwell D, Cave C, et al. Clinical effectiveness and cost-effectiveness of drotrecogin alfa (activated) (Xigris) for the treatment of severe sepsis in adults: a systematic review and economic evaluation2005; 9(11):[1-140 pp.]. Available from: http://cochranelibrary-wiley.com/o/cochrane/cldare/articles/DARE-12005008225/frame.html. |
| 75. Heyland DK, Johnson AP, Reynolds SC, Muscedere J. Procalcitonin for reduced antibiotic exposure in the critical care setting: a systematic review and an economic evaluation (Provisional abstract)2011; 39(7):[1792-9 pp.]. Available from: http://cochranelibrary-wiley.com/o/cochrane/cleed/articles/NHSEED-22011001263/frame.html. |
| 76. Hjelmgren J, Persson U, Tennvall GR. Local treatment pattern versus trial-based data: a cost-effectiveness analysis of drotrecogin alfa (activated) in the treatment of severe sepsis in Sweden2005; 12(5):[425-30 pp.]. Available from: http://cochranelibrary-wiley.com/o/cochrane/cleed/articles/NHSEED-22005006630/frame.html. |
| 77. Jones AE, Troyer JL, Kline JA. Cost-effectiveness of an emergency department-based early sepsis resuscitation protocol2011; 39(6):[1306-12 pp.]. Available from: http://cochranelibrary-wiley.com/o/cochrane/cleed/articles/NHSEED-22011001001/frame.html. |
| 78. Mouncey PR, Osborn TM, Power GS, Harrison DA, Sadique MZ, Grieve RD, et al. Protocolised Management In Sepsis (ProMISe): a multicentre randomised controlled trial of the clinical effectiveness and cost-effectiveness of early, goal-directed, protocolised resuscitation for emerging septic shock2015; (4). Available from: http://cochranelibrary-wiley.com/o/cochrane/clhta/articles/HTA-32010000374/frame.html. |
| 79. Neilson AR, Burchardi H, Schneider H. Cost-effectiveness of immunoglobulin M-enriched immunoglobulin (Pentaglobin) in the treatment of severe sepsis and septic shock2005; 20(3):[239-49 pp.]. Available from: http://cochranelibrary-wiley.com/o/cochrane/cleed/articles/NHSEED-22006006189/frame.html. |
| 80. Reboli AC, Rotstein C, Kett DH, Maschio M, Cartier S, Chambers R, et al. Resource utilization and cost of treatment with anidulafungin or fluconazole for candidaemia and other forms of invasive candidiasis: focus on critically ill patients (Provisional abstract)2011; 29(8):[705-17 pp.]. Available from: http://cochranelibrary-wiley.com/o/cochrane/cleed/articles/NHSEED-22011001268/frame.html. |
| 81. Talmor D, Greenberg D, Howell MD, Lisbon A, Novack V, Shapiro N. The costs and cost-effectiveness of an integrated sepsis treatment protocol (Provisional abstract)2008; 36(4):[1168-74 pp.]. Available from: http://cochranelibrary-wiley.com/o/cochrane/cleed/articles/NHSEED-22008100597/frame.html. |
| 82. Westwood M, Ramaekers B, Whiting P, Tomini F, Joore M, Armstrong N, et al. Procalcitonin testing to guide antibiotic therapy for the treatment of sepsis in intensive care settings and for suspected bacterial infection in emergency department settings: a systematic review and cost-effectiveness analysis (Structured abstract)2015; (4). Available from: http://cochranelibrary-wiley.com/o/cochrane/clhta/articles/HTA-32015001172/frame.html. |
| 83. Sacristán JA, Prieto L, Huete T, Artigas A, Badia X, Chinn C, et al. Cost-effectiveness of drotrecogin alpha in the treatment of severe sepsis in Spain2004; 18(1):[50-7 pp.]. Available from: http://cochranelibrary-wiley.com/o/cochrane/clcentral/articles/161/CN-00470161/frame.html. |
| **Exclusion reason: Letter** |
| 84. Anonymous. Activated protein C, coagulation, imflammation and the treatment of severe sepsis. Nederlands Tijdschrift voor Geneeskunde. 2001;145(45):2196-7. |
| **Exclusion reason: Not economic evaluation** |
| 85. Armen SB, Freer CV, Showalter JW, Crook T, Whitener CJ, West C, et al. Improving Outcomes in Patients With Sepsis. American Journal of Medical Quality. 2016;31(1):56-63. |
| 86. Balk RA, Kadri SS, Cao Z, Robinson SB, Lipkin C, Bozzette SA. Effect of Procalcitonin Testing on Health-care Utilization and Costs in Critically Ill Patients in the United States. Chest. 2017;151(1):23-33. |
| 87. Bansal N, Gopalakrishnan R, Sethuraman N, Ramakrishnan N, Nambi PS, Kumar DS, et al. Experience with beta-D-Glucan assay in the management of critically ill patients with high risk of invasive candidiasis: An observational study. Indian Journal of Critical Care Medicine. 2018;22(5):364-8. |
| 88. Bloomfield R, Noble DW. Corticosteroids for septic shock - A standard of care? British Journal of Anaesthesia. 2004;93(2):178-80. |
| 89. Bloos F, Bayer O, Sachse S, Straube E, Reinhart K, Kortgen A. Attributable costs of patients with candidemia and potential implications of polymerase chain reaction-based pathogen detection on antifungal therapy in patients with sepsis. Journal of Critical Care. 2013;28(1):2-8. |
| 90. Brooks A, Ekleberry A, McMahon J, Begle R, Johnson M, Rizzo J, et al. Evaluation of clinical practice guidelines on outcome of infection in medical intensive care unit patients. Infectious Diseases in Clinical Practice. 1999;8(2):97-106. |
| 91. Chan AL, Hsieh HJ, Lin SJ. Pharmacist intervention in activated protein C therapy for severe sepsis: influence on health and economic outcomes. International Journal of Clinical Pharmacology & Therapeutics. 2009;47(4):229-35. |
| 92. Concia E, Azzini A. Treating bacteremia in critical patients: A long course of combined antimicrobial therapy or a short course with a single antibiotic? Minerva Anestesiologica. 2008;74(5):153-4. |
| 93. Deliberato RO, Marra AR, Sanches PR, Martino MD, Ferreira CE, Pasternak J, et al. Clinical and economic impact of procalcitonin to shorten antimicrobial therapy in septic patients with proven bacterial infection in an intensive care setting. Diagnostic Microbiology & Infectious Disease. 2013;76(3):266-71. |
| 94. Ernst FR, Johnston JA, Pulgar S, He J, Ball DE, Young JK, et al. Timing of drotrecogin alfa (activated) initiation in treatment of severe sepsis: a database cohort study of hospital mortality, length of stay, and costs. Current Medical Research & Opinion. 2007;23(1):235-44. |
| 95. Fernando SM, Rochwerg B, Reardon PM, Thavorn K, Seely AJE, Perry JJ, et al. Emergency Department disposition decisions and associated mortality and costs in ICU patients with suspected infection. Critical Care. 2018;22(1):172. |
| 96. Giamarellos-Bourboulis EJ, Mylona V, Antonopoulou A, Tsangaris I, Koutelidakis I, Marioli A, et al. Effect of clarithromycin in patients with suspected Gram-negative sepsis: results of a randomized controlled trial. Journal of Antimicrobial Chemotherapy. 2014;69(4):1111-8. |
| 97. Gonzalez-Ruiz A, Richardson J. Are glycopeptides still appropriate and convenient for empiric use? Journal of Chemotherapy. 2008;20(5):531-41. |
| 98. Guirgis FW, Jones L, Esma R, Weiss A, McCurdy K, Ferreira J, et al. Managing sepsis: Electronic recognition, rapid response teams, and standardized care save lives. Journal of Critical Care. 2017;40:296-302. |
| 99. Ha YE, Peck KR, Joo EJ, Kim SW, Jung SI, Chang HH, et al. Impact of first-line antifungal agents on the outcomes and costs of candidemia. Antimicrobial Agents & Chemotherapy. 2012;56(7):3950-6. |
| 100. Heimann SM, Cornely OA, Wisplinghoff H, Kochanek M, Stippel D, Padosch SA, et al. Candidemia in the intensive care unit: analysis of direct treatment costs and clinical outcome in patients treated with echinocandins or fluconazole. European Journal of Clinical Microbiology & Infectious Diseases. 2015;34(2):331-8. |
| 101. Hohn A, Schroeder S, Gehrt A, Bernhardt K, Bein B, Wegscheider K, et al. Procalcitonin-guided algorithm to reduce length of antibiotic therapy in patients with severe sepsis and septic shock. BMC Infectious Diseases. 2013;13:158. |
| 102. Hutchison RW, Govathoti DA, Fehlis K, Zheng Q, Cottrell JH, Franklin N, et al. Improving severe sepsis outcomes: cost and time to first antibiotic dose. DCCN - Dimensions of Critical Care Nursing. 2011;30(5):277-82. |
| 103. Hwang TL. Potential Use of Albumin Administration in Severe Sepsis. Journal of the Chinese Medical Association. 2009;72(5):225-6. |
| 104. Ilia S, Briassoulis P, Briassoulis G. Polymyxin B hemoperfusion in septic shock: Nothing overmuch (Meden Agan)! Journal of Thoracic Disease. 2017;9(9):2716-9. |
| 105. Innocenti F, Bianchi S, Guerrini E, Vicidomini S, Conti A, Zanobetti M, et al. Prognostic scores for early stratification of septic patients admitted to an emergency department-high dependency unit. European Journal of Emergency Medicine. 2014;21(4):254-9. |
| 106. Jellison TK, McKinnon PS, Rybak MJ. Epidemiology, resistance, and outcomes of Acinetobacter baumannii bacteremia treated with imipenem-cilastatin or ampicillin-sulbactam. Pharmacotherapy:The Journal of Human Pharmacology & Drug Therapy. 2001;21(2):142-8. |
| 107. Jiang SP, Zhu ZY, Ma KF, Zheng X, Lu XY. Impact of pharmacist antimicrobial dosing adjustments in septic patients on continuous renal replacement therapy in an intensive care unit. Scandinavian Journal of Infectious Diseases. 2013;45(12):891-9. |
| 108. Jones SL, Ashton CM, Kiehne L, Gigliotti E, Bell-Gordon C, Disbot M, et al. Reductions in Sepsis Mortality and Costs After Design and Implementation of a Nurse-Based Early Recognition and Response Program. Joint Commission Journal on Quality & Patient Safety. 2015;41(11):483-91. |
| 109. Judd WR, Stephens DM, Kennedy CA. Clinical and economic impact of a quality improvement initiative to enhance early recognition and treatment of sepsis. Annals of Pharmacotherapy. 2014;48(10):1269-75. |
| 110. Kalil AC. Does recombinant activated protein C work in patients with severe sepsis? Critical Care Medicine. 2010;38(4):1217-20. |
| 111. Kipnis E, Vallet B. Early norepinephrine resuscitation of lifethreatening hypotensive septic shock: It can do the job, but at what cost? Critical Care. 2010;14(450). |
| 112. Koenig A, Picon PD, Feijo J, Silva E, Westphal GA. Estimate of the economic impact of implementing an in hospital protocol for the early detection and treatment of severe sepsis in public and private hospitals in southern Brazil. Revista Brasileira de Terapia Intensiva. 2010;22(3):213-9. |
| 113. Kotapati S, Kuti JL, Nicolau DP. Role of a Clinical Pharmacist on Drotrecogin Alfa (Activated) Outcomes in a Large Community Teaching Hospital. Journal of Infectious Disease Pharmacotherapy. 2003;6(3):55-68. |
| 114. Kron J, Kron S, Wenkel R, Schuhmacher HU, Thieme U, Leimbach T, et al. Extended daily on-line high-volume haemodiafiltration in septic multiple organ failure: a well-tolerated and feasible procedure. Nephrology Dialysis Transplantation. 2012;27(1):146-52. |
| 115. Kuan WS, Ibrahim I, Leong BS, Jain S, Lu Q, Cheung YB, et al. Emergency Department Management of Sepsis Patients: A Randomized, Goal-Oriented, Noninvasive Sepsis Trial. Annals of Emergency Medicine. 2016;67(3):367-78.e3. |
| 116. Kyeremanteng K, Shen J, Thavorn K, Fernando SM, Herritt B, Chaudhuri D, et al. Cost analysis of Omega-3 supplementation in critically ill patients with sepsis. Clinical Nutrition ESPEN. 2018;25:63-7. |
| 117. MacLaren R, Bond CA, Martin SJ, Fike D. Clinical and economic outcomes of involving pharmacists in the direct care of critically ill patients with infections. Critical Care Medicine. 2008;36(12):3184-9. |
| 118. McGee WT, Steingrub JS, Callas PW. Financial impact of pulmonary artery catheterization in severe sepsis. Clinical Intensive Care. 2000;11(3):137-43. |
| 119. Mohr NM, Harland KK, Shane DM, Ahmed A, Fuller BM, Torner JC. Inter-hospital transfer is associated with increased mortality and costs in severe sepsis and septic shock: An instrumental variables approach. Journal of Critical Care. 2016;36:187-94. |
| 120. Patel TS, Kaakeh R, Nagel JL, Newton DW, Stevenson JG. Cost Analysis of Implementing Matrix-Assisted Laser Desorption Ionization-Time of Flight Mass Spectrometry Plus Real-Time Antimicrobial Stewardship Intervention for Bloodstream Infections. Journal of Clinical Microbiology. 2017;55(1):60-7. |
| 121. Perez KK, Olsen RJ, Musick WL, Cernoch PL, Davis JR, Peterson LE, et al. Integrating rapid diagnostics and antimicrobial stewardship improves outcomes in patients with antibiotic-resistant Gram-negative bacteremia. Journal of Infection. 2014;69(3):216-25. |
| 122. Reboli AC, Rotstein C, Kett DH, Maschio M, Cartier S, Chambers R, et al. Resource utilization and cost of treatment with anidulafungin or fluconazole for candidaemia and other forms of invasive candidiasis: focus on critically ill patients. Pharmacoeconomics. 2011;29(8):705-17. |
| 123. Sango A, McCarter YS, Johnson D, Ferreira J, Guzman N, Jankowski CA. Stewardship approach for optimizing antimicrobial therapy through use of a rapid microarray assay on blood cultures positive for Enterococcus species. Journal of Clinical Microbiology. 2013;51(12):4008-11. |
| 124. Serpa Neto A, Schultz MJ, Festic E. Ventilatory support of patients with sepsis or septic shock in resource-limited settings. Intensive Care Medicine. 2016;42(1):100-3. |
| 125. Shorr AF, Micek ST, Jackson WL, Jr., Kollef MH. Economic implications of an evidence-based sepsis protocol: can we improve outcomes and lower costs? Critical Care Medicine. 2007;35(5):1257-62. |
| 126. Silverman LZ, Hoesel LM, Desai A, Posa P, Purtill MA, Brandt MM. It takes an intensivist. American Journal of Surgery. 2011;201(3):320-3. |
| 127. Taylor B, Burns D, van de Wal BW, T MB, Keeton GR. Should health care money in South Africa be spent on drotrecogin alfa? South African Medical Journal. 2003;93(7):500-1. |
| 128. Thomas G, Balk EM, Jaber BL. Effect of Intensive Insulin Therapy and Pentastarch Resuscitation on Acute Kidney Injury in Severe Sepsis. American Journal of Kidney Diseases. 2008;52(1):13-7. |
| 129. Via G, Storti E, Spreafico A, Melniker L, Neri L. Point of care ultrasound for sepsis management in resource-limited settings: Time for a new paradigm for global health care. Intensive Care Medicine. 2012;38(8):1405-7. |
| 130. Wilke MH, Grube RF, Bodmann KF. The use of a standardized PCT-algorithm reduces costs in intensive care in septic patients - a DRG-based simulation model. European Journal of Medical Research. 2011;16(12):543-8. |
| 131. Yu DT, Platt R, Lanken PN, Black E, Sands KE, Schwartz JS, et al. Relationship of pulmonary artery catheter use to mortality and resource utilization in patients with severe sepsis. Critical Care Medicine. 2003;31(12):2734-41. |
| 132. Zilberberg MD, Kollef MH, Arnold H, Labelle A, Micek ST, Kothari S, et al. Inappropriate empiric antifungal therapy for candidemia in the ICU and hospital resource utilization: a retrospective cohort study. BMC Infectious Diseases. 2010;10:150. |
| 133. Zilberberg MD, Nathanson BH, Sulham K, Fan W, Shorr AF. Carbapenem resistance, inappropriate empiric treatment and outcomes among patients hospitalized with Enterobacteriaceae urinary tract infection, pneumonia and sepsis. BMC Infectious Diseases. 2017;17(1):279. |
| 134. Leisman DE, Doerfler ME, Ward MF, Masick KD, Wie BJ, Gribben JL, et al. Survival Benefit and Cost Savings From Compliance With a Simplified 3-Hour Sepsis Bundle in a Series of Prospective, Multisite, Observational Cohorts. Critical Care Medicine. 2017;45(3):395-406. |
| 135. Bouza E, Bustinza A, Caliz B, Escribano P, Fernandez-Cruz A, Fernandez-Quero J, et al. Antifungal stewardship in a tertiary-care institution: A bedside intervention. Clinical Microbiology and Infection. 2015;21(5):492.e1-.e9. |
| 136. Huang SW, Guan XD, Chen J, Ou Yang B. Clinical study and long-term evaluation of immunomodulation therapy on trauma, severe sepsis and multiple organ dysfunction syndrome patients. Zhongguo Wei Zhong Bing Ji Jiu Yi Xue/Chinese Critical Care Medicine/Zhongguo Weizhongbing Jijiuyixue. 2006;18(11):653-6. |
| 137. Caputo PP, Zuccon W, Faccini M, Manelli A, Bonandrini L. Iatrogenic abdominal sepsis in biliary tract surgery. [Italian] Le sepsi addominali iatrogene nella chirurgia delle vie biliari. Chirurgia. 2001;14(6):197-204. |
| 138. Tsalis K. Drotrecogin alfa (activated) in severe sepsis. Medical Science Monitor. 2004;10(10):LE19. |
| 139. Walsh GL, Chiasson P, Hedderich G, Wexler MJ, Meakins JL. The open abdomen. The Marlex mesh and zipper technique: a method of managing intraperitoneal infection. Surgical Clinics of North America. 1988;68(1):25-40. |
| 140. Verma RK, Bhattacharyya P, Sen MR, Kumari G. Comparative efficacy of cefpirome & ceftazidime alone or in combination with isepamycin in empiric treatment of septicaemia in patients admitted to ICU. Journal of Anaesthesiology Clinical Pharmacology. 2004;20(4):369-77. |
| **Exclusion reason: Not sepsis** |
| 141. Auzinger G, Playford EG, Graham CN, Knox HN, Weinstein D, Kantecki M, et al. Cost-effectiveness analysis of anidulafungin for the treatment of candidaemia and other forms of invasive candidiasis. BMC Infectious Diseases. 2015;15:463. |
| 142. Niederman MS, Chastre J, Solem CT, Wan Y, Gao X, Myers DE, et al. Health economic evaluation of patients treated for nosocomial pneumonia caused by methicillin-resistant Staphylococcus aureus: Secondary analysis of a multicenter randomized clinical trial of vancomycin and linezolid. Clinical Therapeutics. 2014;36(9):1233-43.e1. |
| 143. Rotstein C, Cragin L, Laverdiere M, Garber G, Bow EJ, Scalera A, et al. Economic evaluation of voriconazole for the treatment of candidemia in Canadian adults. Canadian Journal of Infectious Diseases and Medical Microbiology. 2008;19(3):219-26. |
| 144. Scheetz MH, Bolon MK, Postelnick M, Noskin GA, Lee TA. Cost-effectiveness analysis of an antimicrobial stewardship team on bloodstream infections: A probabilistic analysis. Journal of Antimicrobial Chemotherapy. 2009;63(4):816-25. |
| **Exclusion reason: Review article** |
| 145. Alexander SL, Ernst FR. Use of drotrecogin alfa (activated) in older patients with severe sepsis. Pharmacotherapy:The Journal of Human Pharmacology & Drug Therapy. 2006;26(4):533-8. |
| 146. Anonymous. Drotrecogin alfa (activated) for severe sepsis. Drug & Therapeutics Bulletin. 2006;44(1):5-8. |
| 147. Banks SM, Gerstenberger E, Eichacker PQ, Natanson C. Long-term cost effectiveness of drotrecogin alfa (activated): An unanswered question. Critical Care Medicine. 2003;31(1):308-9. |
| 148. Burchell M, Ruiz F, Barnett D. Appraisal of treatment for severe sepsis in intensive care units. Annals of the Royal College of Surgeons of England. 2005;87(4):284. |
| 149. Chalupka AN, Talmor D. The economics of sepsis. Critical Care Clinics. 2012;28(1):57-76. |
| 150. Chan CM, Shorr AF. Dollars and sense in sepsis. Critical Care Medicine. 2011;39(6):1559-60. |
| 151. Dasta JF, Cooper LM. Impact of drotrecogin alfa (activated) on resource use and implications for reimbursement. Pharmacotherapy:The Journal of Human Pharmacology & Drug Therapy. 2002;22(12 Pt 2):216S-22S. |
| 152. Durthaler JM, Ernst FR. Evidence-based disease management in medication-use evaluation: application to drotrecogin alfa (activated) in severe sepsis. American Journal of Health-System Pharmacy. 2006;63(15):1453-60. |
| 153. Ebm CC, Sutton L, Rhodes A, Cecconi M. Cost-effectiveness in goal-directed therapy: Are the dollars spent worth the value? Journal of Cardiothoracic and Vascular Anesthesia. 2014;28(6):1660-6. |
| 154. Frampton JE, Foster RH. Drotrecogin alfa (activated): a pharmacoeconomic review of its use in severe sepsis. Pharmacoeconomics. 2004;22(7):445-76. |
| 155. Haley M, Cui X, Minneci PC, Deans KJ, Natanson C, Eichacker PQ. Recombinant human activated protein C in sepsis: Previous concerns and current usage. Therapy. 2004;1(1):123-9. |
| 156. Hussain AM. Drotrecogin alfa (activated) in severe sepsis and septic shock. Anaesthesia, Pain and Intensive Care. 2011;15(2):79-80. |
| 157. Mayr A, Aigner M, Lass-Florl C. Anidulafungin for the treatment of invasive candidiasis. Clinical Microbiology & Infection. 2011;17(Suppl 1):1-12. |
| 158. Parrillo JE. Severe sepsis and therapy with activated protein C. New England Journal of Medicine. 2005;353(13):1398-400. |
| 159. Rice TW, Bernard GR. Drotrecogin alfa (activated) for the treatment of severe sepsis and septic shock. American Journal of the Medical Sciences. 2004;328(4):205-14. |
| 160. Schumacher HK, Muller-Nordhorn J, Roll S, Willich SN, Greiner W. Efficacy and effectiveness of recombinant human activated protein C in severe sepsis of adults. GMS Health Technology Assessment. 2007;3:Doc05. |
| 161. Vanscoy GJ. Management challenge with drotrecogin alfa (activated). American Journal of Health-System Pharmacy. 2002;59(Suppl 1):S23-9. |
| 162. Wiedemann HP. Activated protein C was cost-effective for prolonging survival in a subgroup of patients with severe sepsis. ACP Journal Club. 2003;138(3):81. |
| 163. Piacevoli Q, Palazzo F, Azzeri F. Cost evaluation of patients with severe sepsis in intensive care units. Minerva Anestesiologica. 2004;70(6):453-71. |
| 164. Tebas P, Badia X, Segu L, Garcia Alonso F, Rovira J. Cost-efficacy analysis of antiendotoxin monoclonal antibody treatment in sepsis by gram negative bacteria. Medicina Clinica. 1993;101(19):757-8. |
| 165. Wood JG. Antiendotoxin monoclonal antibodies for the treatment of gram-negative sepsis syndrome and gram-negative shock. Cancer Bulletin. 1992;44(6):525-7. |
| 166. Chalfin DB, Teres D, Rapoport J. A price for cost-effectiveness: Implications for recombinant human activated protein C (rhAPC). Critical Care Medicine. 2003;31(1):306-8. |
| 167. Torrabadella de Reynoso P, Salgado Remigio A. New treatments in severe sepsis. Scientific, economic and ethical aspects. Medicina Clinica. 1999;113(1):18-9. |
| 168. Berto P, Ronco C, Cruz D. Cost-effectiveness analysis of polymyxin-b immobilized fiber column and conventional medical therapy in the management of abdominal septic shock in italy. PharmacoEconomics - Italian Research Articles. 2012;14(2):144-5. |
| 169. Wiedermann CJ. Perspectives in economic analyses of sepsis. Wiener Klinische Wochenschrift. 2002;114(15-16):660-2. |
| 170. Anonymous. Cost-effective of activated drotrecogin alfa in the treatment of severe sepsis. Modern Aspects in Sepsis. 2002;1(1):12-3. |
