# Supplementary material for: Health economic evaluations of sepsis interventions in critically ill adult patients: a systematic review
Source: J Intensive Care. 2020 Jan 8;8:5. doi: 10.1186/s40560-019-0412-2 (PMC6950865; doi:10.1186/s40560-019-0412-2)
Supplement: Supplementary file 3 — Additional file 3. Tables A3-1 to A3-3 Characteristics and results of included evaluations [file 40560_2019_412_MOESM3_ESM.docx]

**Additional file 3. Characteristics and results of included evaluations**

**Additional file for:**

Higgins AM, Brooker J, Mackie M, Cooper DJ and Harris A

Health economic evaluations of sepsis interventions in critically ill adult patients: a systematic review

**Table A3-1** Characteristics of included evaluation

**Table A3-2** Main economic evaluation results, included septic shock subgroup results

**Table A3-3** Additional results of included evaluations – included costs, total costs, effectiveness outcomes and sub-group results

**Table A3-1** **Characteristics of included evaluation**

| **First author Publication year** | **Study design** | **Funding source** | **Target population** | **Setting** | **Intervention** | **Comparator** | **Perspective** | **Currency** | **Time**  **horizon** | **Discount rate** | **Quality score** |
| --- | --- | --- | --- | --- | --- | --- | --- | --- | --- | --- | --- |
| **Antibiotic therapy economic evaluations** | | | | | | | | | | | |
| Yakovlev 2006 | Cost  -effectiveness;  Trial-based (randomised comparative trial) | Not stated | Severe sepsis - nosocomial pneumonia and intra-abdominal infections | ICU (18 centres across 15 cities),  Russia | Meropenem | Standard Care - standard antibiotic therapy | Not stated (appears to be hospital) | Ruble 2005-2006 | Not stated, (appears to be hospitalisation) | N/A | 12/20 (60%) |
| Zilberberg  2009 | Cost-effectiveness and cost-utility;  Model | Pharmaceutical industry (Astellas Pharma US Inc.) | Sepsis with suspected ICU-acquired candidemia | ICU,  USA | 100 mg daily empiric micafungin | 400 mg daily fluconazole | Base case: Hospital  Reference case: Societal. | USD 2008 | Base case: Hospitalisation  Reference case: Lifetime | Base case: N/A  Reference case: 3% for costs (not stated for outcomes) | 20/23 (87%) |
| Berto 2011 | Cost-effectiveness;  Trial-based (RCT; retrospective CEA) and model | Pharmaceutical industry (unrestricted grant from ESTOR SpA) | Severe sepsis or septic shock post emergency surgery for intra-abdominal infection | ICU (10 tertiary care centres),  Italy | Polymyxin-B Immobilized Fiber Column plus Standard Care | Standard Care | Hospital | Euro 2010 | Lifetime | 3% (N/A for costs) | 19/21 (90%) |
| Scawn 2012 | Cost minimisation analysis;  Trial-based (Pilot RCT) | National Institute for Health Research Health Technology Assessment Programme | Suspected sepsis and commenced on surviving sepsis care bundle antibiotics | ICU and postoperative CCU, single-centre,  UK | 7 days of antibiotics | 2 days of antibiotics | NHS Hospital Level | GBP, year not stated | Not stated (appears to be hospitalisation) | N/A | 16.5/21 (79%) |
| Tsaganos 2016 | Cost-effectiveness;  Trial-based (RCT) | Hellenic Institute for the Study of Sepsis | Ventilator-associated pneumonia and sepsis | ICU, multicenter,  Greece | Intravenous clarithromycin for three consecutive days | Placebo | Not stated (appears to be hospital) | Euro 2002 | Not stated (appears to 90 days) | N/A | 10/19 (53%) |

| **First author Publication year** | **Study design** | **Funding source** | **Target population** | **Setting** | **Intervention** | **Comparator** | **Perspective** | **Currency** | **Time**  **horizon** | **Discount rate** | **Quality score** |
| --- | --- | --- | --- | --- | --- | --- | --- | --- | --- | --- | --- |
| **Procalcitonin algorithms – economic evaluations** | | | | | | | | | | | |
| Harrison 2015 | Cost-utility;  Model | Not stated | Suspected sepsis | ICU,  USA | Procalcitonin-guided treatment algorithm | Standard Care | Hospital | Currency not stated (appears to be USD), 2014 | 1 year | N/A | 17/23 (74%) |
| Kip 2015 | Cost-effectiveness;  Model | Pharmaceutical industry (BRAHMS GbH, part of Thermo Fisher Scientific, Germany) | Sepsis | ICU,  Netherlands | Procalcitonin algorithm for discontinuation of antibiotic therapy | Standard Care | Hospital | Euro 2013 | Hospitalisation | N/A | 18/21 (86%) |
| Westwood 2015 | Cost- utility;  Model | National Institute for Health Research HTA programme | Suspected or confirmed sepsis | ICU,  UK | Procalcitonin testing to guide antibiotic therapy | Standard Care | Not stated (appears to be healthcare payer) | GBP, year not stated | 6 months | N/A | 18/23 (78%) |
| **Fluid interventions - economic evaluations** | | | | | | | | | | | |
| Guidet 2007 | Cost-effectiveness; Model | Pharmaceutical industry (Laboratoire Franc¸ais du Fractionnement et des Biotechnologies) | Severe sepsis with circulatory, renal or respiratory failure. | ICU,  France | Albumin | Standard Care | French National Health System | Euro, year not stated | Not stated  (appears to be lifetime) | Not stated | 11/23 (48%) |
| Farrugia 2014 | Cost-effectiveness;  Model | Not stated | Severe sepsis (network meta-analysis also specified septic shock) | Not stated,  USA | Albumin | Crystalloid, Hydroxyethyl starch | US third party payer | USD 2012 | Not stated (appears to be lifetime) | Not stated | 16/22 (73%) |

| **First author Publication year** | **Study design** | **Funding source** | **Target population** | **Setting** | **Intervention** | **Comparator** | **Perspective** | **Currency** | **Time**  **horizon** | **Discount rate** | **Quality score** |
| --- | --- | --- | --- | --- | --- | --- | --- | --- | --- | --- | --- |
| **Immunoglobulin therapy economic evaluations** | | | | | | | | | | | |
| Neilson 2005 | Cost-effectiveness;  Model | Pharmaceutical industry (Biotest Pharma GmbH) | Severe sepsis and septic shock | ICU,  Germany | Immunoglobulin M–enriched immunoglobulin (Pentaglobin) plus Standard Care | Standard Care | Hospital | Euro, year not specified | ICU stay | N/A | 17/22 (77%) |
| Soares 2012 and 2014 | Cost-utility;  Model | National Institute for Health Research HTA programme | Severe sepsis and septic shock | ICU (mixed medical/surgical),  UK | Intravenous immunoglobulin (IVIG) plus Standard Care | Standard Care | NHS and Personal Social Services | GBP 2009 | Lifetime (30 years) | 3.5% | 22/24 (92%) |
| **Early goal-directed therapy and other sepsis protocol economic evaluations** | | | | | | | | | | | |
| Huang 2007 | Cost-effectiveness and cost-utility; Model | National Institute of Health | Severe sepsis and septic shock | ED,  USA | EGDT (ED-based strategy, Mobile team strategy, ICU-based strategy) | Standard Care | Hospital and Societal (Reference Case) | USD 2005 | Hospital case: Hospitalisation  Reference case: Lifetime | 3% | 21/24 (88%) |
| Talmor 2008 | Cost-effectiveness and cost-utility;  Trial-based (cohort with historical controls) and model | Not stated | Intervention group: - septic shock  Control group - infection evidenced by blood culture order | ED and ICU (single centre),  USA | Multiple Urgent Sepsis Therapies (MUST) protocol – incorporates EGDT, antibiotics, tight glycaemic, low todal volume ventilation in ALI, control assessment for drotrecogin alfa (activated) and steroids | Standard Care | Healthcare system | USD 2004 | Lifetime | 3% (N/A for costs) | 18/21 (86%) |
| Jones 2011 | Cost-effectiveness and cost-utility;  Trial-based (prospective before and after study) and model | National Institutes of Health/National Institute of General Medical Sciences | Septic shock | ED (Single centre),  USA | EGDT | Standard Care | Not stated – (appears to be Hospital) | USD 2006 | Not stated (appears to be lifetime) | 3% (N/A for costs) | 17/21 (81%) |

| **First author Publication year** | **Study design** | **Funding source** | **Target population** | **Setting** | **Intervention** | **Comparator** | **Perspective** | **Currency** | **Time**  **horizon** | **Discount rate** | **Quality score** |
| --- | --- | --- | --- | --- | --- | --- | --- | --- | --- | --- | --- |
| Suarez 2011 | Cost-effectiveness and cost-utility;  Trial-based (observational before and after study) and model | Instituto de Salud Carlos III, Ministerio de Ciencia e Innovacion  FEDER program, European Commission | Severe sepsis or septic shock | 59 medical-surgical ICU, multicentre (59 medical-surgical ICUs), Spain | Surviving Sepsis Campaign Protocol education program | Standard Care | Health-care system | Euro 2006 | Lifetime | 3% (N/A for costs) | 19/22 (86%) |
| Assuncao 2014 | Cost-effectiveness; Trial based (prospective cohort study involving a historical comparison) and model | Not stated | Severe sepsis or septic shock | ICU (private hospital),  Brazil | Managed protocol (based on Surviving Sepsis Campaign recommendations), including lectures, e-learning modules and explanatory brochures | Standard Care | Not stated (appears to be Hospital) | USD 2010 | Lifetime | 0% (N/A for costs) | 13.5/21 (64%) |
| Noritomi 2014 | Cost-utility;  Trial-based (observational before and after study) and model | Not stated | Severe sepsis and septic shock | Private hospitals, multicentre,  Brazil | Mulitfaceted sepsis education program to improve compliance with Surviving Sepsis Campaign bundle (full compliance with bundle) | Usual care - no compliance with bundle | Healthcare system | USD  year not specified | Not stated (appears to be lifetime) | 3% (N/A for costs) | 18/22 (82%) |
| Mouncey 2015 (2 publications) | Cost-utility;  Trial-based (RCT) and model | UK National Institute for Health Research Health Technology Assessment Programme | Septic shock, within 6 hours of presentation to ED | NHS Hospitals,  UK | EGDT | Standard Care | Health and personal health services | GBP 2012-13 | 90 days,  1 year (primary)  20 years (lifetime analysis) | 3.5%  (lifetime analysis) | 21/22 (95%) |
| PRISM 2017 | Cost-utility;  Trial-based (patient-level meta-analysis of data from 3 trials: ProCESS, ARISE, ProMISe) and model | US National Institute of General Medical Sciences, National Institutes of Health; NHMRC; ICF; Alfred foundation; UK NIHR HTA Programme | Septic shock;  Inclusion in ARISE, PROMISE of ProCESS trial; | ED and ICU, Multinational (138 hospitals in USA, Australia, NZ, Finland, Hong Kong, Republic of Ireland, England) | EGDT | Standard Care | Health-services | USD 2012-13 | 90 days | N/A | 18/23 (78%) |
| **First author Publication year** | **Study design** | **Funding source** | **Target population** | **Setting** | **Intervention** | **Comparator** | **Perspective** | **Currency** | **Time**  **horizon** | **Discount rate** | **Quality score** |
| **Pathogen identification – economic evaluations** | | | | | | | | | | | |
| Lehmann 2010 | Cost-effectiveness and cost-utility; Model | Pharmaceutical industry (Roche Diagnostics) | Sepsis | ICU,  International | Polymerase chain reaction microbiological diagnosis | Standard Care (including blood cultures) | Healthcare payer | Euro, year not stated | Not stated | Not stated | 8/24 (33%) |
| Alvarez 2012 | Cost minimisation;  Trial-based (retrospective observational) | Not stated | Severe sepsis or septic shock | ICU,  Spain | Polymerase chain reaction microbiological diagnosis | Standard Care | Hospital | Euro 2006 | Not stated (appears to be hospitalisation) | N/A | 12/20 (60%) |
| Stevenson 2016 | Cost-utility;  Model | National Institute for Health Research HTA programme | Suspected sepsis | ICU,  UK | LightCycler SeptiFast testing MGRADE®, SepsiTest™,  IRIDICA BAC BSI assay | Standard Care | NHS and Personal Social Services perspective | GBP, year not stated | Lifetime | 3.5% | 21/23 (91%) |
| Cambau 2017 | Cost-effectiveness; Trial-based (cluster crossover RCT) | French Ministry of Health | Severe sepsis and septic shock | Hospital, multicentre,  France | LightCycler®SeptiFast testing | Standard diagnostic workup | Hospital | Euro 2013 | 30 days | N/A | 12/19 (63%) |
| **Other interventions – economic evaluations** | | | | | | | | | | | |
| Champunot 2014 | Cost-effectiveness;  Model | Not stated | Severe sepsis or septic shock | Hospital, multicentre,  Thailand | Initial ICU admission (following admission to ED) | Standard Care (delayed admission to ICU; admitted to ward post-ED) | Hospital | Baht 2012 | Not stated (appears to be hospitalisation) | N/A | 12/22 (55%) |
| Ward 2016 | Cost-utility;  Model | Pharmaceutical industry (Abbott Point of Care) | Suspected sepsis | ED,  USA | Point of care lactate testing | Standard Care | Not stated (reported as societal in discussion but does not appear to be societal) | Not stated | Not stated (appears to be lifetime) | 3% (N/A for costs) | 15/23 (65%) |

| **Interventions no longer in clinical practice** | | | | | | | | | | | |
| --- | --- | --- | --- | --- | --- | --- | --- | --- | --- | --- | --- |
| **First author Publication year** | **Study design** | **Funding source** | **Target population** | **Setting** | **Intervention** | **Comparator** | **Perspective** | **Currency** | **Time**  **horizon** | **Discount rate** | **Quality score** |
| **Monoclonal antibodies – economic evaluations** | | | | | | | | | | | |
| Schulman 1991 | Cost-effectiveness;  Model | Not stated | Sepsis (no description of criteria) | Hospital,  USA | HA-1A monoclonal antibody | Placebo | Societal | Not stated | Lifetime | 5% | 14/22 (64%) |
| Barriere 1992 | Cost-effectiveness;  Model | Not stated | Gram negative sepsis and septic shock | Hospital (does not specify ICU),  USA | HA-1A monoclonal antibody | Standard Care | Hospital | USD 1990-91 | Lifetime | Not stated (N/A for costs) | 3/23 (13%) |
| Badia 1993 | Cost-effectiveness;  Model | Pharmaceutical industry (Centocor Incorporated) | Sepsis and septic shock | ICU,  Spain | HA-1A monoclonal antibody | Standard Care | Not stated (appears to be hospital) | Pesetas; 1988 | Lifetime | 5% | 17/23 (74%) |
| Chalfin 1993 | Cost-effectiveness;  Model | Pharmaceutical industry (partially supported by a grant from Pfizer-Roerig, NY) | Sepsis with suspected gram-negative infection, not in refractory shock | ICU,  USA | E5 or HA-1A monoclonal antibody | Standard Care | Hospital | Not stated | Not stated (appears to be 30 days) | N/A | 17/22 (77%) |
| Chang 1993 | Cost-effectiveness;  Trial-based (non-randomised open label study) | Not stated | Sepsis, non-cardiac surgery | ICU ,  Single centre,  UK | HA-1A monoclonal antibody | Standard Care | Not stated (appears to be Hospital) | Not stated (appears to be GBP 1989-1990) | Not stated | N/A | 6/20 (30%) |
| Linden 1995 | Cost-effectiveness;  Trial-based (cohort study) and model | Not stated | Sepsis and suspected gram-negative bacteremia | ICUs (multiple ICUs within one medical centre),  USA | HA-1A monoclonal antibody | Placebo | Model 1: Societal  Model 2: Hospital  Model 3: Not stated | Not stated | Not stated | 5% (model 1 -Schulman analysis); not stated for other models | 15.5/23 (67%) |
| Wang 1999 | Cost-effectiveness;  Model | Not stated | Gram-negative sepsis in patients with NRTI or CUTI | Hospital,  USA | E5 or HA-1A monoclonal antibody | Standard Care | Healthcare provider | USD; year not stated | 28 days | N/A | 13/22 (59%) |

| **Interventions no longer in clinical practice** | | | | | | | | | | | |
| --- | --- | --- | --- | --- | --- | --- | --- | --- | --- | --- | --- |
| **First author Publication year** | **Study design** | **Funding source** | **Target population** | **Setting** | **Intervention** | **Comparator** | **Perspective** | **Currency** | **Time**  **horizon** | **Discount rate** | **Quality score** |
| **Drotrecogin alfa (activated) economic evaluations** | | | | | | | | | | | |
| Manns 2002 | Cost-effectiveness and cost-utility;  Model | NFP  Foundation | Severe sepsis; admitted to ICU | ICU,  Canada (3 centres) | Drotrecogin alfa  (activated); | Conventional care | Healthcare payer | USD 2001 | Lifetime | 5% | 19/24 (79%) |
| Angus 2003 | Cost-effectiveness and cost-utility;  Trial-based (RCT) and model | Pharmaceutical industry | Severe sepsis (meeting criteria within a 24 hour window) | Multinational (164 hospitals, 11 countries), USA costs | Drotrecogin alfa (activated); | Placebo | Societal | USD 2000 | Base case: 28 days  Reference case: Lifetime | Base case: N/A  Reference case: 3% | 21/22 (95%) |
| Betancourt 2003 | Cost-effectiveness; Model | No funding source declared (authors declare no pharmaceutical funding was used) | Severe sepsis with one or more organ failures | Level 1 trauma centre,  USA | Drotrecogin alfa (activated) plus Standard Care | Standard Care | Hospital (level 1 trauma centre) | USD 2002 | 28 days | N/A | 18/22 (82%) |
| Fowler 2003 | Cost-effectiveness and cost-utility;  Model | No external  funding | Severe sepsis | ICU,  USA | Drotrecogin alfa (activated) | Usual therapy | Societal | USD 2001 | Lifetime | 3% | 21/24 (88%) |
| Neilson 2003 and 2004 | Cost-effectiveness; Model | Pharmaceutical industry (Eli Lilly & Co. Ltd) | Severe sepsis | ICU,  Germany | Drotrecogin alfa (activated) plus Standard Care | Standard Care | Healthcare payer | Euro 1998-9 | Lifetime. | 0% (N/A for costs; outcomes 3% in sensitivity analysis) | 20/23 (87%) |
| Sacristan 2004 | Cost-effectiveness; Model | Not stated | Severe sepsis | ICU,  Spain | Drotrecogin alfa (activated) | Standard Care | Health care payer | Euro 2001 | Not stated (appears to be lifetime) | 0% (N/A for costs; outcomes 3% and 5% in sensitivity analysis) | 17/23 (74%) |
| Davies 2005 | Cost-effectiveness and Cost-utility; Model | Pharmaceutical industry (Eli Lilly and Co Ltd) | Severe sepsis with multiple organ failure | ICU,  UK | Drotrecogin alfa (activated) | Placebo | National Health Service (Health care payer) | GBP 2002 | Not stated  (appears to be lifetime) | 1.5% (N/A for costs) | 19/24 (79%) |
| Hjelmgren 2005 | Cost-effectiveness and cost-utility; Model | Not stated | Septicemia infection with at least 1 acute organ dysfunction | ICU,  Sweden | Drotrecogin alfa (activated) plus Standard Care | Standard Care | Not stated (appears to be Hospital) | Euro 2002 | Not stated; (appears to be lifetime) | 3% (N/A for costs) | 16/24 (67%) |
| **Interventions no longer in clinical practice** | | | | | | | | | | | |
| **First author Publication year** | **Study design** | **Funding source** | **Target population** | **Setting** | **Intervention** | **Comparator** | **Perspective** | **Currency** | **Time**  **horizon** | **Discount rate** | **Quality score** |
| Franca 2006 | Cost-effectiveness and cost-utility; Model | Pharmaceutical industry (Lilly, France) | Patients with severe sepsis and multi organ failure | ICU, France | Drotrecogin alfa (activated) plus Standard Care | Standard Care | Not stated | USD 2002 (converted from Euro) | Lifetime | 0% (N/A for costs; outcomes 5% in sensitivity analysis) | 15/24 (63%) |
| Green 2005 and 2006 | Cost-effectiveness and cost-utility; Model | UK NHS R&D Health Technology Assessment Programme | Severe sepsis | NHS hospitals, UK | Drotrecogin alfa (activated) plus Standard Care | Standard Care | Healthcare payer (NHS in England and Wales) | GBP, year not stated | Not stated (appears to be lifetime) | 3.5% for long-term NHS cost, 6% for future costs, 1.5% for future benefits (life years) | 19/24 (79%) |
| Costa 2007 | Cost-effectiveness; Model | Not stated; one author is a funded scholar of les Fonds de la Recherche en Sante du Quebec | Severe sepsis with ≥ 1 organ dysfunction | Not stated, (Canada) | Drotrecogin alfa (activated) | Standard Care | Health care payer | USD 2006 | 20 years | 3% | 19/24 (79%) |
| Dhainaut 2007 | Cost-effectiveness and cost-utility;  Trial-based (prospective observational study) and model | French Ministry of Health | Severe sepsis with ≥ 2 organ failures | ICU, France | Drotrecogin alfa (activated) | Standard Care | Healthcare provider | Euro 2004 | Not stated (appears to be lifetime) | 0% (N/A for costs) | 16/22 (67%) |
| Sadique 2011 | Cost-utility;  Trial-based (cohort) and model | Not stated | Severe sepsis with multiple organ failures | ICU,  UK | Drotrecogin alfa (activated) | Standard Care | Not stated (appears to be Hospital) | GBP 2010-2011 | Lifetime | 3.5% | 17/22 (77%) |

CUTI=community-acquired urinary tract infection; ED=emergency department; EGDT=early goal-directed therapy; GBP=British pound sterling; ICU=intensive care unit; N/A=not applicable; NHS=National Health Service; NRTI=nosocomial respiratory tract infection; RCT=randomised controlled trial; UK=United Kingdom; USA=United States of America; USD=United States Dollars

**Table A3-2 Main economic evaluation results, included septic shock subgroup results**

| **First author Publication year** | **Cost/life saved**  **Local currency *(2018 USD)*** | **Cost/life year gained**  **Local currency *(2018 USD)*** | **Cost/QALY**  **Local currency *(2018 USD)*** | **Probability of cost-effectiveness** | **Other** |
| --- | --- | --- | --- | --- | --- |
| **Antibiotic therapy economic evaluations** | | | | | |
| Yakovlev 2006 | NR | NR | NR | NR | Meropenem cost-effectiveness ratio: 694 rubles/patient recovery *($68)*  Standard care cost-effectiveness ratio: 1531 rubles/patient recovery *($150)* |
| Zilberberg 2009 | $61,446/LS *($70,663)* | $22,230/LYG *($25,565)* | $34,734/QALY *($39,944)* | NR | NR |
| Berto 2011 | NR | $3,864/LYG *($5,797)* | NR | 97.8% probability cost-effective at WTP €60,000/LYG | NR |
| Scawn 2012 | NR | NR | NR | NR | Mean antibiotic cost per patient:  2-day group £168.97 *($265)*  7-day group: £375.86 *($589)* |
| Tsaganos 2016 | NR | NR | NR | NR | Day 25:  Placebo: €14,701.10/patient staying alive *($31,044)*  Clarithromycin: €13,100.50/patient staying alive *($27,664)*  Day 45:  Placebo: €26,249.50/patient staying alive *($55,431)*  Clarithromycin: €19,303.10/patient staying alive *($40,953)* |
| **Procalcitonin algorithms economic evaluations** | | | | | |
| Harrison 2015 | NR | NR | Dominant | 67.3% probability cost-effective at WTP $0/QALY  69.4% probability cost-effective at WTP $50,000/QALY  71.1% probability cost-effective at a WTP $100,000/QALY | NR |
| Kip 2015 | NR | NR | NR | NR | €2,043/antibiotic day avoided *($2,764)* |
| Westwood 2015 | NR | NR | Low risk patients: Dominant, High risk patients: Dominant | Low risk patients: 97% probability cost-effective at WTP £20,000/QALY  High risk patients: 95% probability cost-effective at WTP £20,000/QALY | NR |

| **First author Publication year** | **Cost/life saved**  **Local currency *(2018 USD)*** | **Cost/life year gained**  **Local currency *(2018 USD)*** | **Cost/QALY**  **Local currency *(2018 USD)*** | **Probability of cost-effectiveness** | **Other** |
| --- | --- | --- | --- | --- | --- |
| **Fluid therapy economic evaluations** | | | | | |
| Guidet 2007 | €6,037/LS *($8,211)* | €617/LYG *($839)* | NR | NR | NR |
| Farrugia 2014 | NR | Dominant over hydroxyethyl starch | NR | NR | Cost-effectiveness ratios:  Albumin $9,253/LYG *($10,178)*; probability cost-effective at $10,000/LYG 56%  Hydroxyethyl starch $24,363/LYG *($26,799)*; probability cost-effective at $10,000/LYG <5%  Crystalloid $10,036/LYG *($11,040)* probability cost-effective at $10,000/LYG 40% |
| **Immunoglobulin therapy economic evaluations** | | | | | |
| Neilson 2005 | €10,565/LS *($15,738)* | NR | NR | 56.3% probability cost-effective at WTP €12,000/LS  83.9% probability cost-effective at WTP €15,000/LS | NR |
| Soares 2012 | NR | NR | £20,850/QALY *($34,362)* | 50.5% probability cost-effective at WTP £20,000/QALY  78.9% probability cost-effective at WTP £30,000/QALY | NR |
| **Early goal-directed therapy and other sepsis protocol economic evaluations** | | | | | |
| Huang 2007 | Dominant (ED, ICU and mobile team) | NR | ED team: $7,019/QALY *($9,125)*  ICU team: $2,749/QALY *$3,574)*  Mobile team: $6,931/QALY *($9,010)* | Hospital perspective:  99.5% (ED, 99.4%; team, 99.3%; ICU, 99.8%) probability dominant (cost savings and better outcome)  Societal perspective:  ED 97.5%; team 97.7%; ICU 96.7% probability cost-effective at WTP $20,000/QALY | NR |
| Talmor 2008 | NR | $11,264/LYG *($14,981)* | $16,309/QALY *($21,691)* | 72.6% probability cost-effective at a WTP $50,000/QALY  78.0% probability cost-effective at a WTP $100,000/QALY | NR |
| Jones 2011 | NR | $4,667/LYG *($5,787)* | $5,397/QALY *($6,692)* | 97% probability cost-effective at WTP $20,000/QALY  98% probability cost-effective at a WTP $50,000/QALY | NR |
| Suarez 2011 | €48,039/LS *($80,852)* | €4,435/LYG *($7,464)* | €6,428/QALY *($10,819)* | 96.5% probability cost-effective at WTP €30,000/LYG  95.6% probability cost-effective at WTP €30,000/QALY | NR |
| **First author Publication year** | **Cost/life saved**  **Local currency *(2018 USD)*** | **Cost/life year gained**  **Local currency *(2018 USD)*** | **Cost/QALY**  **Local currency *(2018 USD)*** | **Probability of cost-effectiveness** | **Other** |
| Assuncao 2014 | Dominant | NR | NR | NR | NR |
| Noritomi 2014 | NR | NR | -$5,383/QALY (dominant)  *(-$5,706)* | NR | NR |
| Mouncey 2015 | NR | NR | NR | 90 day time horizon:  <20% probability cost-effective at all WTP from £0/QALY to £100,000/QALY  1 year time horizon:  <30% probability cost-effective at WTP £20,000/QALY  20 year time horizon:  <50% probability cost-effective at all WTP from £0/QALY to £100,000/QALY | 90 day time horizon:  INB (at £20,000/QALY): -£1,000 *(-$1,553)*  1 year time horizon:  INB (at £20,000/QALY): -£725  *(-$1,126)*  20 year time horizon:  INB (at £20,000/QALY): -£1446 *(-$2,246)* |
| PRISM 2017 | NR | NR | NR | <25% probability cost-effective at all WTP from $0/QALY to $500,000/QALY | Incremental Net Benefit (at $20,000/QALY) –  ProCESS: -$1,266 *(-$1,367)*  ARISE: -$2,032 *(-$2,195)*  ProMISe: -$1,172 *(-$1,266)* |
| **Pathogen identification economic evaluations** | | | | | |
| Lehmann 2010 | €11,477/LS *($16,789)* | NR | €3,107/QALY *($4,545)* | NR | NR |
| Alvarez 2012 | NR | NR | NR | NR | Net saving: €9970/patient *($16,780)* |
| Stevenson 2016 | NR | NR | Base case 1:  Purchase machinery and no purchase of machinery  SeptiFast: Dominated  SepsiTest: Dominated  IRIDICA: Dominated  Base case 2:  Mortality rate 13% or 29%, between 2.4 and 68 blood samples per day and machinery needing to be purchased or not  SeptiFast: ranging from £1,463/QALY *($2,199)* to £9,862/QALY *($14,822)*  SepsiTest: Dominant  IRIDICA: ranging from dominant to £5,264/QALY *($7,911)* | NR | NR |
| Cambau | NR | NR | NR | NR | Reported as weak dominance (ICER/microbial documentation not reported) |
| **First author Publication year** | **Cost/life saved**  **Local currency *(2018 USD)*** | **Cost/life year gained**  **Local currency *(2018 USD)*** | **Cost/QALY**  **Local currency *(2018 USD)*** | **Probability of cost-effectiveness** | **Other** |
| **Other interventions - economic evaluations** | | | | | |
| Champunot 2014 | 45,307 baht/LS *($4,029)* | NR | NR | NR | NR |
| Ward 2016 | NR | NR | $33,318/QALY *($34,984)* [reported as $31,590/QALY in abstract *($33,170*)] | NR | NR |

$=United States dollar; ED=emergency department; ICER=incremental cost effectiveness ratio; ICU=intensive care unit; INB=Incremental Net Benefit; LS=life saved; LYG=life year gained; N/A=not applicable; NR=not reported; QALY=quality-adjusted life years; RCT=randomised controlled trial; RTI=respiratory tract infection; USD=United States Dollars; UTI=urinary tract infection; WTP=willingness to pay

| **Interventions no longer in clinical practice** | | | | | |
| --- | --- | --- | --- | --- | --- |
| **First author Publication year** | **Cost/life saved**  **Local currency *(2018 USD)*** | **Cost/life year gained**  **Local currency *(2018 USD)*** | **Cost/QALY**  **Local currency *(2018 USD)*** | **Probability of cost-effectiveness** | **Other** |
| **Monoclonal antibodies economic evaluations** | | | | | |
| Schulman 1991 | $104,600/LS (reported as per death averted) *($193,510)* | $24,100/LYG *($44,585)* | NR | NR | NR |
| Barriere 1992 | $205,178/LS *($379,579)*  Gram negative septic shock subgroup: $28,950/LS  *($53,558)* | Ranging from $10,250/LYG *($18,963)* for 20 year survival time to $410,356/LYG (*$759,159)* for 0.5 year survival time  Gram negative septic shock subgroup:  Ranging from $1,448/LYG *($2,679)* for 20 year survival time to $57,900/LYG *($107,115)* for 0.5 year survival time | NR | NR | NR |
| Badia 1993 | NR | 859,288ptas/LYG *($1,830,283)*  Septic shock subgroup: $293,810ptas/LYG *($625,815)* | NR | NR | NR |
| Chalfin 1993 | Monoclonal antibody cost $2,000: $14,125/LS *($24,719)*  Monoclonal antibody cost $4,000: $39,125/LS *($68,469)* | NR | NR | NR | NR |
| Chang 1993 | NR | NR | NR | NR | Effective cost per survivor HA1A: £45,500 *($118,502)*  Effective cost per survivor control: £42,300 *($110,167)* |

| **Interventions no longer in clinical practice** | | | | | |
| --- | --- | --- | --- | --- | --- |
| **First author Publication year** | **Cost/life saved**  **Local currency *(2018 USD)*** | **Cost/life year gained**  **Local currency *(2018 USD)*** | **Cost/QALY**  **Local currency *(2018 USD)*** | **Probability of cost-effectiveness** | **Other** |
| Linden 1995 | Model 1 basecase: Ineffective  Model 1 adjusted: $90,400/LS *($149,160)*  Model 2 (decreased morbidity) basecase: Infinity/LS  Model 2 (decreased morbidity) adjusted: $84,325/LS *($139,136)*  Model 2 (same morbidity)  basecase: Infinity/LS  Model 2 (same morbidity)  adjusted: $124,625/LS *($205,631)*  Model 3 ($2000 HA1A): $61,600/LS *($101,640)*  Model 3 ($4000 HA1A): $101,600/LS *($167,640)* | NR | NR | NR | NR |
| Wang 1999 | NR | NR | NR | NR | Community-acquired UTI:  At $2000 HA1A: $30,000/ additional resolution of major complications *($45,600)*  At $4000 HA1A: $60,303/ additional resolution of major complications *($91,661)*  Nosocomial RTI:  At $2000 HA1A: $17,049/ additional resolution of major complications *($25,914)*  At $4000 HA1A: $34,562/ additional resolution of major complications *($52,524)* |

| **Interventions no longer in clinical practice** | | | | | |
| --- | --- | --- | --- | --- | --- |
| **First author Publication year** | **Cost/life saved**  **Local currency *(2018 USD)*** | **Cost/life year gained**  **Local currency *(2018 USD)*** | **Cost/QALY**  **Local currency *(2018 USD)*** | **Probability of cost-effectiveness** | **Other** |
| **Drotrecogin alfa (activated) economic evaluations** | | | | | |
| Manns 2002 | NR | $27,936/LYG *($39,669)* | $46,560/QALY *($66,115)* | 86% probability cost-effective at WTP $50,000/QALY | NR |
| Angus 2003 | $160,000/LS *($233,600)*  Septic shock subgroup: $133,800/LS *($195,348)* | $33,300/LYG *($48,618)* | $48,800/QALY *($71,248)*  Septic shock subgroup:  $33,700/QALY *($49,202)* | 84.7% probability cost-effective at WTP $200,000/LS;  97.9% probability cost-effective at WTP $500,000/LS  89.1% probability cost-effective at WTP $100,000/LYG  82% probability cost-effective at WTP $100,000/QALY | NR |
| Betancourt 2003 | ≥1 organ failure:$104,100/LS  *($145,740)*  ≥2 organ failures: $78,075/LS  *($109,305)*  ≥3 organ failures: $69,578/LS  *($97,409)*  ≥4 organ failures: $56,727/LS  *($79,418)* | NR | NR | NR | NR |
| Fowler 2003 | $129,262/LS *($183,552)* | $15,801/LYG *($22,437)* | $20,047/QALY *($28,467)* | NR | NR |
| Neilson 2003 | NR | €14,119/LYG (undiscounted)  *($22,493)*  €17,723/LYG (discounted)  *($28,234)* | NR | NR | NR |
| Sacristan 2004 | NR | €13,550/LYG  *($25,743)* | NR | NR |  |
| Davies 2005 | NR | £4,608/LYG (PROWESS data)  *($9,351)*  £7,625/LYG (EVBI data) *($15,474)* | £6,679/QALY (PROWESS data) *($13,554)*  £11,051/QALY (EVBI data)  *($22,426)* | NR | NR |
| Hjelmgren 2005 | NR | Local Unit Price Approach: €21,556/LYG *($3,206)*  Local Data Approach: €18,126/LYG *($2,696)*  Local Data-with Trial Age Structure: €21,401/LYG *($3,183)* | Local Unit Price Approach: €31,241/QALY *($4,646)*  Local Data Approach: €26,232/QALY *($3,901)*  Local Data with Trial Age Structure: €31016/QALY *($4,613)* | NR | NR |
| Franca 2006 | NR | $11,812/LYG *($16,537)* | $19,686/QALY *($27,560)* | NR | NR |
| Green 2006 | NR | £5495/LYG *($9,773)* | £9161/QALY *($16,293)* | 96.8% probability cost-effective at WTP £20,000/QALY |  |

| **Interventions no longer in clinical practice** | | | | | |
| --- | --- | --- | --- | --- | --- |
| **First author Publication year** | **Cost/life saved**  **Local currency *(2018 USD)*** | **Cost/life year gained**  **Local currency *(2018 USD)*** | **Cost/QALY**  **Local currency *(2018 USD)*** | **Probability of cost-effectiveness** | **Other** |
| Costa 2007 | NR | NR | NR | 48% probability cost-effective at WTP $30,000/LYG  59% probability cost-effective at WTP $50,000/LYG |  |
| Dhainaut 2007 | NR | €20,278/LYG *($28,814)* | €33,797/QALY *($48,025)* | 74.5% probability cost-effective at WTP €50,000/LYG  64.3% probability cost-effective at WTP €50,000/QALY |  |
| Sadique 2011 | NR | NR | £30,158/QALY *($47,839)* | 5% probability cost-effective at WTP £20,000/QALY | INB (at £20,000/QALY):  -£5934 *(-$9,413)* |

$=United States dollar; ED=emergency department; ICER=incremental cost effectiveness ratio; ICU=intensive care unit; INB=Incremental Net Benefit; LS=life saved; LYG=life year gained; N/A=not applicable; NR=not reported; QALY=quality-adjusted life years; RCT=randomised controlled trial; RTI=respiratory tract infection; USD=United States Dollars; UTI=urinary tract infection; WTP=willingness to pay

**Table A3-3** **Additional results of included evaluations – included costs, total costs, effectiveness outcomes and sub-group results**

| **First author**  **Publication year** | **Included costs** | **Total costs** | **Outcomes** | **Subgroup cost-effectiveness results *(2018 USD)*** |
| --- | --- | --- | --- | --- |
| **Antibiotic therapy economic evaluations** | | | | |
| Yakovlev 2006 | Intervention costs: Antibiotics (trial and additional)  Hospitalisation costs: ICU (daily cost) | Meropenem: 55,907 ruble  Standard care: 71,349 ruble | Meropenem:  Patient recovery rate 80.6%  APACHE II 15-20 Mortality 3.3%  APACHE II 21-25 Mortality 35.5%  Standard care:  Patient recovery rate 46.6%  APACHE II 15-20 Mortality 7.1%  APACHE II 21-25 Mortality 36.7% | APACHE II 15-20  Meropenem cost-effectiveness ratio: 561 ruble/patient recovery *($55)*  Standard care cost-effectiveness ratio: 1183 ruble/patient recovery *($116)*  APACHE II 21-25  Meropenem cost-effectiveness ratio :1056 ruble/patient recovery *($104)*  Standard care cost-effectiveness ratio: 2447 ruble/patient recovery *($240)* |
| Zilberberg 2009 | Intervention costs: Antibiotics  Hospitalisation costs: Nil specified  Post-hospitalisation costs: Age-specific annual health care costs | Not reported | Not reported | None reported |
| Berto 2011 | Intervention costs: PMX-F  Hospitalisation costs: ICU days (including catecholamine, RRT and ventilation days), ward days | PMX-F: €59,922  Standard care: €42,712 | PMX-F:  28 day mortality 32%  LYs 9.37 years  Standard care:  28 day mortality 53%  LYs 4.92 years | None reported |
| Scawn 2012 | Intervention costs: Antibiotic costs  Hospitalisation costs: ICU days (although not included in cost-minimisation analysis) | 2-day group: £168.97  7-day group: £375.86 | N/A – cost minimisation | None reported |
| Tsaganos 2016 | Intervention costs: Antimicrobials  Hospitalisation costs: ICU days, ward days, radiology tests, interventions (i.e.,  catheterisations, tracheostomies, and hemodialysis) and respective consumables, laboratory tests (including blood cell counting, biochemistry, blood gas, and microbiology), antifungals, and non-antimicrobial drugs | Not reported | Clarithromycin:  28 day mortality 31%  9 day mortality 43%  Placebo:  28 day mortality 28%  90 day mortality 60% | None reported |

| **First author**  **Publication year** | **Included costs** | **Total costs** | **Outcomes** | **Subgroup cost-effectiveness results *(2018 USD)*** |
| --- | --- | --- | --- | --- |
| **Procalcitonin algorithms economic evaluations** | | | | |
| Harrison 2015 | Intervention costs: PCT testing  Hospitalisation costs: Antimicrobials, daily costs for septicemia, nephrotoxicity, profound nephrotoxicity and clostridium difficile infection | PCT: $40,597  Standard care: $40,663 | PCT:  QALYs 0.9187  Standard care:  QALYs 0.9185 | None reported |
| Kip 2015 | Intervention costs: PCT test  Hospitalisation costs: diagnostic tests (blood cultures, other routinely performed laboratory tests), ICU days, ventilation days, dialysis days, ward days, IV antibiotic days | PCT: €34,414  Standard care: €37,917 | PCT:  Antibiotic days 9.9 days  Standard care:  Antibiotic days 11.6 days | None reported |
| Westwood 2015 | Intervention costs: PCT test  Hospitalisation costs: Antibiotic days, ICU days, ward days | PCT:  Low risk patients £26,622  High risk patients £42,602  Standard care:  Low risk patients £29,890  High risk patients £45,464 | PCT:  Low risk patients  Mortality Not reported  LYs 0.391 years  QALYs 0.256 years  High risk patients  Mortality Not reported  LYs 0.389 years  QALYs 0.254 years  Standard care:  Low risk patients  Mortality 16.9%  LYs 0.390 years  QALYs 0.254 years  High risk patients  Mortality 22.2%  LYs 0.388 years  QALYs 0.252 years | Low risk patients: dominant; 97% probability cost-effective at WTP £20,000/QALY  High risk patients: dominant; 95% probability cost-effective at WTP £20,000/QALY |
| **Fluid therapy economic evaluations** | | | | |
| Guidet 2007 | Intervention costs: Albumin  Hospitalisation costs: ICU days and DRG cost | Albumin: €26,263 (25,295) + €218  Standard care: €26,263 (25,295) | Albumin:  Mortality 49.1%  Life expectancy 9.78 years  Standard care:  Mortality 53.7%  Life expectancy 9.78 years | None reported |

| **First author**  **Publication year** | **Included costs** | **Total costs** | **Outcomes** | **Subgroup cost-effectiveness results *(2018 USD)*** |
| --- | --- | --- | --- | --- |
| Farrugia 2014 | Intervention costs: Albumin, crystalloid or Hydroxyethyl starches cost  Hospitalisation costs: Hospitalisation, Complications - continuous renal replacement therapy, bleeding treatment, transfusion support | Albumin: $20,403  Crystalloid: $20,133  Hydroxyethyl starches: $28,091 | Albumin:  Mortality 31.2%  Life expectancy 2.21 years  Crystalloid:  Mortality 33.27%  Life expectancy 2.00 years  Hydroxyethyl starches:  Mortality 35.8%  Life expectancy 1.15 years | None reported |
| **Immunoglobulin therapy economic evaluations** | | | | |
| Neilson 2005 | Intervention costs: Pentaglobin  Hospitalisation costs: ICU costs (basic costs, staff, hotel, sepsis therapy, blood therapy, ventilation therapy, renal therapy) stratified by survival | Immunoglobulin: €24,747  Standard care: €22,711 | Pentaglobin:  Mortality 25.06%  Standard care: Mortality 44.34% | None reported |
| Soares 2012 | Intervention costs: IVIG  Hospitalisation costs: ICU days, ward days  Post hospitalisation costs: annual NHS costs post discharge | IVIG: £54,901  Standard care: £45,593 | IVIG:  QALYs 4.35 years  Standard care:  QALYs 3.90 years | 1 organ failure: £26,049/QALY *($42,930);* 26.4% probability cost-effective at WTP £20,000/QALY; 64.8% probability cost-effective at WTP £30,000/QALY  ≥2 organ failures: £20,706/QALY *($34,125);* 51.1% probability cost-effective at WTP £20,000/QALY; 79.3% probability cost-effective at WTP £30,000/QALY  2 organ failures: £21,817/QALY *($35,956);* 45.7% probability cost-effective at WTP £20,000/QALY; 76.3% probability cost-effective at WTP £30,000/QALY  3 organ failures: £20,611/QALY *($33,968*); 51.5% probability cost-effective at WTP £20,000/QALY; 79.5% probability cost-effective at WTP £30,000/QALY  4 organ failures: £22,163/QALY *($36,526);* 43.0% probability cost-effective at WTP £20,000/QALY; 75.6% probability cost-effective at WTP £30,000/QALY  5 organ failures: £26,268/QALY *($43,291);* 22.0% probability cost-effective at WTP £20,000/QALY; 63.3% probability cost-effective at WTP £30,000/QALY  APACHE II: Ranging from £19,868/QALY *($32,744)* for APACHE II=23 (with 54.9% probability cost-effective at WTP £20,000/QALY) to £67,522/QALY *($111,280)* for APACHE II=1 (with 0% probability cost-effective at WTP £20,000/QALY) |

| **First author**  **Publication year** | **Included costs** | **Total costs** | **Outcomes** | **Subgroup cost-effectiveness results *(2018 USD)*** |
| --- | --- | --- | --- | --- |
| **Early goal-directed therapy and other sepsis protocol economic evaluations** | | | | |
| Huang 2007 | Intervention costs: EGDT staff time (and training time), screening costs (ABG and lactate), equipment acquisition, quality assurance  Hospitalisation costs: ICU days (with and without ventilation), intermediate care days, ward days, packed red blood cells, PAC, CVC, arterial line, vasopressor use  Post-hospitalisation costs: lifetime healthcare costs (from National Health Medical Expenditure survey, age-specific) plus nursing home  (charges converted to costs using cost to charge ratio) | EGDT:  Hospital case $37,873  Reference case $78,370  Standard care:  Hospital case $29,157  Reference case $75,196 | Incremental Mortality:  ED team: -16%  ICU team: -12.6%  Mobile team: -16%  Incremental QALYs:  ED team: 0.59 (0.27) years  ICU team: 0.47 (0.26) years  Mobile team: 0.59 (0.27) years | ED team: $7,019/QALY *($9,125)*  ICU team: $2,749/QALY *($3,574)*  Mobile team: $6,931/QALY *($9,010)* |
| Talmor 2008 | Intervention costs: Nil  Hospitalisation costs: In-hospital treatment costs (using hospital accounting system) | MUST protocol: $38,569 ($38,261)  Standard care: $29,762 ($29,006) | MUST protocol:  Mortality 20.3%  LYs 6.128 (6.125) years  QALYs 4.228 (4.227) years  Standard care:  Mortality 29.4%  LYs 5.346 (5.341) years  QALYs 3.689 (3.689) years | None reported |
| Jones 2011 | Intervention costs: Nil other than included in hospitalisation costs  Hospitalisation costs: Hospital costs (based on hospital accounting system)  (charges converted to costs) | EGDT: $20,289 ($19,403)  Standard care: $13,261 ($13,893) | EGDT:  1 year mortality 37%  LYs 7.2 (9.3) years  QALYs 6.4 (5.95) years  Standard care:  1 year mortality 49%  LYs 5.7 (9.2) years  QALYs 5.1 (5.98) years | None reported |
| Suarez 2011 | Intervention costs: SCC protocol drugs, SCC protocol non-pharmacological interventions  Hospitalisation costs: Emergency visits, ICU days, surgical and medical ward days | SSC protocol: €18,671 (€20,792)  Standard care: €16,935 (€18,525) | SSC protocol:  Mortality 39.7%  LYs 5.98 (6.11) years  QALYs 4.12 (4.22) years  Standard care:  Mortality 44.0%  LYs 5.44 (6.05) years  QALYs 3.75 (4.18) years | APACHE II <21: €3,618/LYG *($6,089);* €5,245/QALY *($8,828)*  APACHE II ≥21: €8,909/LYG *($14,994);* €12,915/QALY *($21,736)*  Age <66: €1,505 *($2,533)/*LYG; €2,180/QALY *($3,669)*  Age ≥66: €13,188/LYG *($22,196);* €19,120/QALY *($32,180)*  Subgroup results also available by gender |
| Assuncao 2014 | Intervention costs: Nil other than included in hospitalisation costs  Hospitalisation costs: Full hospitalisation and ICU stay (from hospital charging system) post sepsis diagnosis | Managed protocol: $202,160 ($367,253)  Standard care: $230,759 ($299,424) | Managed protocol:  Mortality 38.4%  LYs 12.0 (14.0) years  Standard care:  Mortality 56.5%  LYs 8.8 (13.3) years | None reported |
| Noritomi 2014 | Intervention costs: Nil  Hospitalisation costs: In-hospital treatment costs | Full compliance with bundle: $16,190  No compliance with bundle: $23,577 | Full compliance with bundle:  Mortality 20%  QALYs 4.43 years  No compliance with bundle:  Mortality 44%  QALYs 3.06 years | Severe sepsis with lactate<4 mmol/L: -$9,938/QALY  *(-$10,534)* (dominant)  Septic shock or severe sepsis and lactate ≥ 4 mmol/L: -$5,074/QALY *(-$5,378)* (dominant) |
| Mouncey 2015 | Intervention costs: Equipment, consumables and blood products in protocol, staff time for delivering protocol, protocol delivery time and location  Hospitalisation costs: ED hours, ICU days (by organ failure), ward days  Post-hospitalisation costs: Hospital readmissions, hospital outpatient and community services use | EGDT:  90 day time horizon: £12,414 (£14,970)  1 year time horizon: £15,139 (£18,345)  20 year time horizon: £33,620 (£25,012)  Standard care:  90 day time horizon: £11,424 (£15,727)  1 year time horizon: £14,375 (£19,179)  20 year time horizon: £32,142 (£25,798) | EGDT:  90 day time horizon:  Mortality 29.5%  QALYs 0.054 (0.048) years  1 year time horizon:  Mortality 40.1%  QALYs 0.352 (0.323)  20 year time horizon:  QALYs 4.584 (3.546)  Standard care:  90 day time horizon:  Mortality 29.2%  QALYs 0.054 (0.048) years  1 year time horizon:  Mortality 41.8%  QALYs 0.351 (0.329)  20 year time horizon:  QALYs 4.582 (3.720) | 90 day time horizon:  MEDS score 0-4: INB (at £20,000/QALY): -£2089 *(-$3,245)*  MEDS score 5-6: INB (at £20,000/QALY): -£2652 *(-$4,120)*  MEDS score 7-9: INB (at £20,000/QALY): £351 *($545)*  MEDS score 10-20: INB (at £20,000/QALY): -£377 *(-$586)*  Age 18-56: INB (at £20,000/QALY): -£3265 *(-$5,072)*  Age 57-67: INB (at £20,000/QALY): -£329 *(-$511)*  Age 68-77: INB (at £20,000/QALY): £1444 *($2,243)*  Age 78-95: INB (at £20,000/QALY): -£2,296 *(-$3,567)*  1 year time horizon:  MEDS score 0-4: INB (at £20,000/QALY): -£1,670 *(-$2,594)*  MEDS score 5-6: INB (at £20,000/QALY): -£2,241 *(-$3,481)*  MEDS score 7-9: INB (at £20,000/QALY): £1,806 *($2,805)*  MEDS score 10-20: INB (at £20,000/QALY): -£1,551 *(-$2,409)*  Age 18-56: INB (at £20,000/QALY): -£3422 *(-$5,316)*  Age 57-67: INB (at £20,000/QALY): £238 *($370)*  Age 68-77: INB (at £20,000/QALY): £1357 *($2,108)*  Age 78-95: INB (at £20,000/QALY): -£1226 *(-$1,904)*  20 year time horizon:  MEDS score 0-4: INB (at £20,000/QALY): -£560 *(-$870)*  MEDS score 5-6: INB (at £20,000/QALY): -£985 *(-$1,530)*  MEDS score 7-9: INB (at £20,000/QALY): £4788 *($7,438)*  MEDS score 10-20: INB (at £20,000/QALY): -£7077 *(-$10,993)*  Age 18-56: INB (at £20,000/QALY): -£2957 *(-$4,593)*  Age 57-67: INB (at £20,000/QALY): £915 *($1,421)*  Age 68-77: INB (at £20,000/QALY): -£2069 *(-$3,214)*  Age 78-95: INB (at £20,000/QALY): £5787 *($8,990)*  Subgroup results also available at each time point for degree of protocolised resuscitation in usual-resuscitation group, SOFA score, time from ED presentation to randomisation and an adherence adjusted analysis, |
| PRISM 2017 | Intervention costs: Equipment, consumables and blood products in protocol, staff time for delivering protocol, protocol delivery time and location  Hospitalisation costs: ED hours, ICU days, Ward days | EGDT:  ProCESS $32,178 ($30,181)  ARISE $25,014 ($25,737)  ProMISe $14,112 ($15,120)  Standard care:  ProCESS $30,930 ($30,150)  ARISE $22,973 ($22,822)  ProMISe $12,906 ($16,017) | EGDT:  Mortality 24.9%  QALYs 0.058 (0.048) years  Standard care:  Mortality 25.4%  QALYs 0.058 (0.048) years | INB (at $20,000/QALY)  Age <57:  ProCESS: $110 *($119)*  ARISE: -$2,989 *(-$3,228)*  ProMISe: -$2,495 *(-$2,695)*  Age 57-71:  ProCESS: $2,648 *($2,860)*  ARISE: -$451 *(-$487)*  ProMISe: $42 *($45)*  Age ≥72:  ProCESS: $1,438 *($1,553)*  ARISE: -$1,660 *(-$1,793)*  ProMISe: -$1,167 *(-$1,260)*  INB (at $20,000/QALY)  APACHE II <14:  ProCESS: -$806 *(-$870)*  ARISE: -$1,401 *(-$1,513)*  ProMISe: $86 *($93)*  APACHE II 14-19:  ProCESS: -$1653 *(-$1,785)*  ARISE: -$2248 *(-$2,428)*  ProMISe: -$761 *(-$822)*  APACHE II ≥20:  ProCESS: -$3,180 *(-$3,434)*  ARISE: -$3,775 *(-$4,077)*  ProMISe: -$2,288 *(-$2,471)*  Subgroup results also available for sex, site of infection, inclusion criterion met, last lactate before randomisation, APACHE II acute physiology score, SOFA score, customised risk of death, invasive mechanical ventilation, vasopressor infusion, and care delivery characteristics, |
| **Pathogen identification economic evaluations** | | | | |
| Lehmann 2010 | Intervention costs: Polymerase chain reaction test  Hospitalisation costs: Not specified | Not reported | Polymerase chain reaction:  Mortality 26.03%  Standard care:  Mortality 32.88% | None reported |
| Alvarez 2012 | Intervention costs: LightCycler SeptiFast (LSC) test  Hospitalisation costs: ICU days, ward days, antibiotics | LCS: €32,228  Standard care: €42,198 | N/A – cost minimisation | None reported |
| Stevenson 2016 | Intervention costs: Tests (SeptiFast, SepsiTest, and IRIDICA), including machinery purchase  Hospitalisation costs: Blood cultures, antimicrobials, ICU days, ward days | Incremental cost  Base case 1:  Purchase of machinery or not  SeptiFast: £201.23 to £205.54  SepsiTest: £142.48 to £149.53  IRIDICA: £270.89 to £314.61  Base case 2:  Mortality rate 13% or 29%, between 2.4 and 68 blood samples per day and machinery needing to be purchased or not  SeptiFast: £64,107 to £652,257  SepsiTest: -£1,476,739 to -£15,963  IRIDICA: -£827,626 to £73,501 | Incremental QALYs  Base case 1:  SeptiFast: 0.00 years  SepsiTest: 0.00 years  IRIDICA: 0.00 years  Base case 2:  Mortality rate 13% or 29%, between 2.4 and 68 blood samples per day and machinery needing to be purchased or not  SeptiFast: 6.88 to 435.50 years  SepsiTest: 9.72 to 615.30 years  IRIDICA: 13.96 to 833.51 years | None reported |
| Cambau 2017 | Intervention costs: LightCycler SeptiFast test  Hospitalisation costs: Diagnostic tests, antibiotic prescriptions, average national cost for DRG (combined with LOS) | LCS: €19,329 (€16,355)  Standard care: €20,995 (€17,593) | LCS:  Microbiologically documented infection 42.6%  Standard care:  Microbiologically documented infection 28.1% | None reported |
| **Other interventions - economic evaluations** | | | | |
| Champunot 2014 | Intervention costs: Not specified  Hospitalisation costs: Hospitalisation, staff and treatments | Immediate ICU group:  37,194 (70,813) baht  Delayed ICU group:  26,275 (58,601) baht | Immediate ICU group:  Mortality 22.2%  Delayed ICU group:  Mortality 46.3% | None reported |
| Ward 2016 | Intervention costs: equipment and training costs (fixed); equipment and staffing costs per patient (variable)  Hospitalisation costs: physician, ICU and ward costs (additional costs for death)  (charges converted to costs using cost to charge ratio) | POC lactate program: $39.53/patient  Usual care: $33.20/patient | POC lactate program:  QALYs 9.7384 years  Usual care:  QALYs 9.7382 years | None reported |

$=United States dollar; APACHE II=Acute Physiology and Chronic Health Evaluation II; ED=emergency department; ICER=incremental cost effectiveness ratio; ICU=intensive care unit; INB=Incremental Net Benefit; IVIG=intravenous immunoglobulin; LCS= LightCycler®Septi*Fast;* LOS=length of stay; LS=life saved; LYG=life year gained; MEDS= Mortality in Emergency Department Sepsis; N/A=not applicable; NHS=National Health Service; PCT=Procalcitonin; PMX-F=polymyxin B immobilized fiber column; POC=point-of-care; QALY=quality-adjusted life years; WTP=willingness to pay

| **Interventions no longer in clinical practice** | | | | |
| --- | --- | --- | --- | --- |
| **First author**  **Publication year** | **Included costs** | **Total costs** | **Outcomes** | **Subgroup cost-effectiveness results *(2018 USD)*** |
| **Monoclonal antibodies economic evaluations** | | | | |
| Schulman 1991 | Intervention costs: HA1A  Hospitalisation costs: Hospitalisation (daily cost) | Incremental cost: $5,650 | Incremental LYG: 0.234 years | Subgroup results available for test strategy and by cost of HA1A |
| Barriere 1992 | Intervention costs: HA1A  Hospitalisation costs: Hospitalisation (including daily costs for gram negative sepsis and septic shock) - costs include hotel, nursing, pharmacy, laboratory, inhalation therapy, surgery and anaethesia, haemodialysis and miscellaneous | Not reported | HA1A:  Mortality 11%  Standard care:  Mortality 13% | Gram negative septicaemia:  $115,178/LS *($213,079)*; Ranging from $5,759/LYG *($10,654)* for 20 year survival time to $230,356/LYG *($426,159)* for 0.5 year survival time  Gram negative septic shock:  $28,950/LS *($53,558)*; Ranging from $1,448/LYG *($2,679)* for 20 year survival time to $57,900/LYG *($107,115)* for 0.5 year survival time |
| Badia 1993 | Intervention costs: HA1A  Hospitalisation costs: ICU (daily cost) | Not reported | HA1A:  Total LYG 497.6 years  Standard care:  Total LYG 437.6 years | Septic shock: 293,810ptas/LYG *($625,796)* |
| Chalfin 1993 | Intervention costs: Biotechnology therapy (E5/HA1A)  Hospitalisation costs: based on charges for retrospective cohort of sepsis patients  (charges not costs reported) | HA1A/E5: $44,438  Standard care: $43,308 | HA1A/E5:  Mortality 38%  Standard care:  Mortality 46% | None reported |
| Chang 1993 | Intervention costs: HA1A  Hospitalisation costs: Cost per TISS point | HA1A: £17,300  Standard care: £15,900 | HA1A:  Mortality 62.1%  Standard care:  Mortality 63% | Gram-negative sepsis:  Effective cost per survivor HA1A: £53,600 *($139,597)*  Effective cost per survivor control: £45,500 *($118,502)* |
| Linden 1995 | Costs as per Schulman 1991, Barriere 1992 and Chalfin 1993 | Not reported | HA1A:  28 day mortality 53%; hospital discharge 63%  Placebo:  28 day mortality 49%; hospital discharge 52% | Subgroup results availably by cost of HA1A |

| **Interventions no longer in clinical practice** | | | | |
| --- | --- | --- | --- | --- |
| **First author**  **Publication year** | **Included costs** | **Total costs** | **Outcomes** | **Subgroup cost-effectiveness results *(2018 USD)*** |
| Wang 1999 | Intervention costs: Biotechnology therapy (E5/HA1A)  Hospitalisation costs: ICU and ward costs, major complications: dialysis for ARF, ventilation for ARDS, blood products for DIC  (charges not costs reported) | Not reported | Not reported | Subgroup results availably by type of infection and cost of HA1A |
| **Drotrecogin alfa (activated) economic evaluations** | | | | |
| Manns 2002 | Intervention costs: Drotrecogin alfa (activated)  Hospitalisation costs: Weekly ICU and hospitalisation cost, bleeding episodes  Post-hospitalisation costs: annual healthcare costs to year 3, constant cost post year 3 | Not reported | Incremental LYG: 0.38 years | APACHE II ≤24: $575,054/LYG *($816,577*); $958,423/QALY *($1,360,961)*  APACHE II >25: $19,723/LYG *($28,007*); $32,872/QALY *($46,678)*  Age <40: $31,158/LYG ($*44*,*244*); $51,930/QALY *($73,741)*  Age 40-59: 25,991/LYG *($36,907*); 43,319/QALY ($*61,513*)  Age 60-79: 27,392/LYG *($38,897*); 45,652/QALY *($64,826*)  Age ≥80: 32,393/LYG *($45,998*); 53,989/QALY *($76,664*) |
| Angus 2003 | Intervention costs: Drotrecogin alfa (activated)  Hospitalisation costs: Hospital costs, physician costs  Post hospitalisation costs: Annual healthcare costs from National Medical Expenditure Survey plus nursing home costs  (charges converted to costs using cost to charge ratio) | Drotrecogin alfa (activated): Base case $47,286  Reference case $156,309  Placebo:  Base case $39,034  Reference case $137,300 | Drotrecogin alfa (activated): Mortality 24.7%  LYs 9.2 years  QALYs 6.3 years  Placebo:  Mortality 30.8%  LYs 8.6 years  QALYs 5.9 years | No shock: $565,200 *($825,192*); dominated ($/QALY)  Shock: 133,800 *($195,348*); 33,700/QALY *($49,202)*  APACHE II 3-19: dominated ($/LS and $/QALY)  APACHE II 20-24: 495,800/LS *($723,868)*; dominated ($/QALY)  APACHE II 25-29: 76,100/LS *($111,106)*; 28400/QALY *($41,464)*  APACHE II 30-53: $98,700/LS *($144,102);* $31,100/QALY *($45,406)*  Age <60: $221,700 *($323,682);* $32,200 *($47,012)*  Age >60: $116,400 *($169,944);* $110,500 *($161,330)*  Subgroup results also available for prior location, co-morbid conditions, median protein C activities, infection type and infection site |
| Betancourt 2003 | Intervention costs: Drotrecogin alfa (activated)  Hospitalisation costs: Hospitalisation (stratified by organ failures and survival), bleeding | Incremental cost:  ≥1 organ failure: $6,246  ≥2 organ failures: $6,246  ≥3 organ failures: $6,262  ≥4 organ failures: $6,240 | Incremental effectiveness (lives saved)  ≥1 organ failure: 0.06  ≥2 organ failures: 0.08  ≥3 organ failures: 0.09  ≥4 organ failures: 0.11 | ≥1 organ failure: $104,100/LS *($145,740)*  ≥2 organ failures: $78,075/LS *($109,305)*  ≥3 organ failures: $69,578/LS *($97,409)*  ≥4 organ failures: $56,727/LS *($79,418)* |

| **Interventions no longer in clinical practice** | | | | |
| --- | --- | --- | --- | --- |
| **First author**  **Publication year** | **Included costs** | **Total costs** | **Outcomes** | **Subgroup cost-effectiveness results *(2018 USD)*** |
| Fowler 2003 | Intervention costs: Drotrecogin alfa (activated)  Hospitalisation costs: Hospitalisation (for one month), bleeding episodes (gastrointestinal bleeding)  Post-hospitalisation costs: Health insurance, medical services, drugs and medical supplies (by age group); Cost of death | Drotrecogin alfa (activated):  $61,751  Standard care:  $51,006 | Drotrecogin alfa (activated):  LYs 8.31 years  QALYs 6.629 years  Standard care:  LYs 7.63 years  QALYs 6.093 years | APACHE II<25: $342,550/LYG *($486,421);* $403,000/QALY *($572,260)*  APACHE II≥25: $10,833/LYG *($15,383);* $13,493/QALY *($19,160)*  Subgroup results also available for protein C deficiency |
| Neilson 2003 | Intervention costs: Drotrecogin alfa (activated)  Hospitalisation costs: ICU days, ICU organ supports (ventilation, vasodilators, renal support, blood therapy (with and without ATIII)) and ward days, | Drotrecogin alfa (activated):  €26,455  Standard care:  €18,125 | Drotrecogin alfa (activated): Mortality 24.1%  Placebo:  Mortality 30.1%  Incremental LYG: 0.47 years | ≥2 organ failures: €10,215/LYG (undiscounted) *($16,273);* €12,880/LYG (discounted) *($20,519)* |
| Sacristan 2004 | Intervention costs: Drotrecogin alfa (activated)  Hospitalisation costs: Hospitalisation (to day 28) | Not reported | Not reported | ≥2 organ failures: €9,800/LYG *($18,619)* |
| Davies 2005 | Intervention costs: Drotrecogin alfa (activated)  Hospitalisation costs: ICU days, ward days | Drotrecogin alfa (activated):  PROWESS data £23,271  EVBI data £22,496  Standard care:  PROWESS data £18,132  EVBI data £17,110 | Drotrecogin alfa (activated):  PROWESS data:  Mortality 26.03%  LYs 10.49 years  QALYs 7.24 years  EVBI data:  Mortality 32.31%  s 9.73 years  QALYs 6.72 years  Standard care:  PROWESS data:  Mortality 33.28%%  LYs 9.38 years  QALYs 6.47 years  EVBI data:  Mortality 36.94%  LYs 9.03 years  QALYs 6.23 years | None reported |

| **Interventions no longer in clinical practice** | | | | |
| --- | --- | --- | --- | --- |
| **First author**  **Publication year** | **Included costs** | **Total costs** | **Outcomes** | **Subgroup cost-effectiveness results *(2018 USD)*** |
| Hjelmgren 2005 | Intervention costs: Drotrecogin alfa (activated)  Hospitalisation costs: ICU and general ward costs to 28 days | Drotrecogin alfa (activated): Local Unit Price Approach: €45,774  Local Data Approach: €45,774  Local Data-with Trial Age Structure: €45,774  Standard care:  Local Unit Price Approach: €63,747  Local Data Approach: €36,073  Local Data-with Trial Age Structure: €36,073 | Incremental LYG:  Local Unit Price Approach: 0.453 years  Local Data Approach: 0.544 years  Local Data-with Trial Age Structure: 0.453 years  Incremental QALYs:  Local Unit Price Approach: 0.313 years  Local Data Approach: 0.375 years  Local Data-with Trial Age Structure: 0.313 years | ≥2 organ failures:  Local Unit Price Approach: €15,965/LYG *($2,374)*; €23,138/QALY *($3,144)*  Local Data Approach: €24,400/LYG *($3,629);* €35,124/QALY *($5,224)*  Local Data-with Trial Age Structure:  €16,410/LYG *($2,440);* €24,661/QALY *($3,668)* |
| Franca 2006 | Intervention costs: Drotrecogin alfa (activated)  Hospitalisation costs: Hospitalisation and ICU costs (investigations, consumables, care staff, indirect costs (hotel services, laundry, pharmacy and administration)) | Incremental cost: $7,545 | Incremental LYG: 0.64 years | <2 organ supports: $17,704/LYG *($24,786);* 29507/QALY *($41,310)*  2 organ supports: $12,942/LYG *($18,119);* $21,570/QALY *($30,198)*  3 organ supports: $7,873/LYG *($11,022);* $13,122/QALY *($18,371)*  Subgroup results also available by admission category |
| Green 2006 | Intervention costs: Drotrecogin alfa (activated)  Hospitalisation costs: ICU days, ward days, bleeding episodes  Post hospitalisation costs: long-term NHS costs | Incremental cost: £6,288 (£593) | Incremental LYG: 1.144 (0.343) years  Incremental QALYs: 0.686 (0.208) years | ≥2 organ failures:£4931/LYG *($8,770);* £8228/QALY *($14,634)*  98.7% probability cost-effective at £20,000/QALY |
| Costa 2007 | Intervention costs: Drotrecogin alfa (activated)  Hospitalisation costs: hospitalisation, bleeding episodes  Post-hospitalisation costs: treatment complications costs years 1 to 3 | Incremental cost: $11,024 | Incremental LYG: 0.344 years | APACHE II≥25: 89% probability ICER ≤$30,000/LYG;  93% probability ICER ≤$50,000/LYG  ≥2 organ failures: 52% probability ICER ≤$50,000/LYG |

| **Interventions no longer in clinical practice** | | | | |
| --- | --- | --- | --- | --- |
| **First author**  **Publication year** | **Included costs** | **Total costs** | **Outcomes** | **Subgroup cost-effectiveness results *(2018 USD)*** |
| Dhainaut 2007 | Intervention costs: Drotrecogin alfa (activated)  Hospitalisation costs: Hospitalisation (ICU and post ICU), based on ICU LOS< Omega score, SAPS II score, and ICU survival | Drotrecogin alfa (activated):  €47,870  Standard care:  €36,717 | Drotrecogin alfa (activated):  Mortality 34.1%  LYs 6.68 (7.33) years  QALYs 4.01 (4.40) years  Standard care:  Mortality 37.4%  LYs 6.13 (7.20) years  QALYs 3.68 (4.32) years | None reported |
| Sadique 2011 | Intervention costs: Drotrecogin alfa (activated)  Hospitalisation costs: ICU days (based on organ failures), Hospital costs  Post-hospitalisation costs: ICU and hospital readmissions (readmission to same ICU) up to 4 years | Drotrecogin alfa (activated):  £36,048 (35,522)  Standard care:  £18,432 (26,708) | Drotrecogin alfa (activated):  Mortality 47.25%  QALYs 5.70 (6.57) years  Standard care:  Mortality 53.04%  QALYs 5.11 (6.61) years | 2 organ failures: dominated; INB (at £20,000/QALY): -£34,031  *(-$53,983)*  3-5 organ failures: £15,561/QALY *($24,684)*; INB (at £20,000/QALY): £5,690 *($9,026)* |

$=United States dollar; APACHE II=Acute Physiology and Chronic Health Evaluation II; ED=emergency department; ICER=incremental cost effectiveness ratio; ICU=intensive care unit; INB=Incremental Net Benefit; IVIG=intravenous immunoglobulin; LCS= LightCycler®Septi*Fast;* LOS=length of stay; LS=life saved; LYG=life year gained; MEDS= Mortality in Emergency Department Sepsis; N/A=not applicable; NHS=National Health Service; PCT=Procalcitonin; PMX-F=polymyxin B immobilized fiber column; POC=point-of-care; QALY=quality-adjusted life years; WTP=willingness to pay
