# Supplementary material for: Health economic evaluations of sepsis interventions in critically ill adult patients: a systematic review
Source: J Intensive Care. 2020 Jan 8;8:5. doi: 10.1186/s40560-019-0412-2 (PMC6950865; doi:10.1186/s40560-019-0412-2)
Supplement: Supplementary file 4 — Additional file 4. CHEERS checklist and assessment of included economic evaluations [file 40560_2019_412_MOESM4_ESM.docx]

**Additional file 4. CHEERS checklist and assessment of included economic evaluations**

**Additional file for:**

Higgins AM, Brooker J, Mackie M, Cooper DJ and Harris A

Health economic evaluations of sepsis interventions in critically ill adult patients: a systematic review

**Note:** Evaluations scoring ≥85% were categorised as having excellent reporting quality, 70 to <85% as very good quality, 55 to <70% as good quality and evaluations scoring <55% were classified as poor quality

**Table A4-1** CHEERS assessment of economic evaluations of antibiotic interventions

| **Author:** | **Yakovlev 2006** | **Zilberberg 2009** | **Berto 2011** | **Scawn 2012** | **Tsaganos 2016** |
| --- | --- | --- | --- | --- | --- |
| **Title** | 1 | 1 | 1 | 0 | ½ |
| **Abstract** | 0 | ½ | 1 | ½ | ½ |
| **Background** | 1 | 1 | 1 | ½ | 1 |
| **Target population** | 1 | 1 | 1 | 1 | 1 |
| **Setting and location** | 1 | ½ | 1 | 1 | 1 |
| **Perspective** | 0 | 1 | 1 | 1 | 0 |
| **Comparators** | 1 | 1 | ½ | 1 | 1 |
| **Time horizon** | 0 | ½ | 1 | ½ | ½ |
| **Discounting** | N/A | ½ | 1 | N/A | N/A |
| **Choice of health outcomes** | 1 | 1 | 1 | 1 | 1 |
| **Measurement of effectiveness** | 1 | 1 | 1 | 1 | 1 |
| **Measurement of preference-based outcomes** | N/A | 1 | N/A | N/A | N/A |
| **Resources and costs** | 1 | 1 | 1 | 1 | 1 |
| **Currency** | ½ | 1 | 1 | ½ | 1 |
| **Choice of model** | N/A | 1 | N/A | ½ | N/A |
| **Assumptions** | N/A | 1 | N/A | ½ | N/A |
| **Analysis** | 1 | ½ | 1 | 1 | ½ |
| **Parameters** | 1 | 1 | 1 | 1 | ½ |
| **Incremental costs and outcomes** | 1 | ½ | 1 | 1 | ½ |
| **Uncertainty** | 0 | 1 | 1 | 1 | 0 |
| **Heterogeneity** | 1 | N/A | 0 | N/A | N/A |
| **Findings** | ½ | 1 | 1 | ½ | ½ |
| **Funding** | 0 | 1 | 1 | 1 | 1 |
| **Conflicts** | 0 | 1 | 1 | 1 | 1 |
| **Total** | **12/20** | **20/23** | **19/21** | **16.5/21** | **10/19** |
| **% score** | **60** | **87** | **90** | **79** | **53** |

0=not reported or insufficiently reported; ½ = reporting unclear or components not reported; 1 = sufficient reporting

**Table A4-2** CHEERS assessment of economic evaluations of fluid therapies, procalcitonin algorithms and immunoglobulins

|  | **Fluid therapy** | | **Procalcitonin algorithms** | | | **Immunoglobulins** | |
| --- | --- | --- | --- | --- | --- | --- | --- |
| **Author:** | **Guidet 2007** | **Farrugia 2014** | **Harrison 2015** | **Kip 2015** | **Westwood 2015** | **Neilson 2005** | **Soares 2012** |
| **Title** | 1 | 1 | 1 | 1 | 1 | 1 | 1 |
| **Abstract** | ½ | 1 | 1 | 1 | ½ | 1 | ½ |
| **Background** | 1 | 1 | 1 | 1 | ½ | 1 | 1 |
| **Target population** | ½ | ½ | 1 | ½ | 1 | ½ | 1 |
| **Setting and location** | 1 | 0 | ½ | 1 | ½ | ½ | 1 |
| **Perspective** | 1 | 1 | 1 | 1 | 0 | 1 | 1 |
| **Comparators** | ½ | 1 | 1 | 1 | 1 | 1 | 1 |
| **Time horizon** | ½ | ½ | 1 | 1 | 1 | ½ | 1 |
| **Discounting** | 0 | ½ |  |  |  | N/A | 1 |
| **Choice of health outcomes** | 1 | 1 | ½ | ½ | 1 | 1 | 1 |
| **Measurement of effectiveness** | 1 | 1 | 1 | 1 | 1 | 1 | 1 |
| **Measurement of preference-based outcomes** | N/A | N/A | ½ | N/A | 1 | N/A | 1 |
| **Resources and costs** | 1 | 1 | 1 | 1 | 1 | 1 | 1 |
| **Currency** | ½ | 1 | ½ | 1 | ½ | 0 | 1 |
| **Choice of model** | 0 | 1 | 1 | ½ | 1 | 1 | 1 |
| **Assumptions** | ½ | 1 | 1 | 1 | 1 | 1 | 1 |
| **Analysis** | ½ | 1 | ½ | 1 | 1 | 1 | 1 |
| **Parameters** | 1 | 1 | 1 | 1 | 1 | 1 | 1 |
| **Incremental costs and outcomes** | ½ | ½ | 1 | 1 | 1 | 1 | 1 |
| **Uncertainty** | 1 | 1 | 1 | 1 | 1 | 1 | 1 |
| **Heterogeneity** | 0 |  | 1 |  | 1 | 1 | 1 |
| **Findings** | 1 | 1 | 1 | 1 | 1 | 1 | ½ |
| **Funding** | 1 | 0 | ½ | 1 | 1 | 1 | 1 |
| **Conflicts** | 0 | 1 | 1 | 1 | 1 | 0 | 1 |
| **Total** | **11/23** | **16/22** | **17/23** | **18/21** | **18/23** | **17/22** | **22/24** |
| **% score** | **48** | **73** | **74** | **86** | **78** | **77** | **92** |

**Table A4-3** CHEERS assessment of economic evaluations of EGDT and other resuscitation protocols

| **Author:** | **Huang 2007** | **Talmor 2008** | **Jones 2011** | **Suarez 2011** | **Assuncao 2014** | **Noritomi 2014** | **Mouncey 2015** | **PRISM 2017** |
| --- | --- | --- | --- | --- | --- | --- | --- | --- |
| **Title** | 1 | 1 | 1 | 1 | 1 | 1 | 1 | 0 |
| **Abstract** | 1 | 1 | ½ | 1 | ½ | ½ | ½ | 0 |
| **Background** | 1 | 1 | 1 | 1 | 1 | 1 | 1 | ½ |
| **Target population** | ½ | 1 | 1 | 1 | 1 | 1 | 1 | 1 |
| **Setting and location** | 1 | 1 | 1 | 1 | 1 | 1 | 1 | 1 |
| **Perspective** | 1 | 1 | 0 | 1 | 0 | 1 | 1 | 1 |
| **Comparators** | 1 | 1 | 1 | 1 | 1 | 1 | 1 | 1 |
| **Time horizon** | ½ | 1 | ½ | ½ | ½ | ½ | 1 | 1 |
| **Discounting** | 1 | 1 | 1 | 1 | 0 | 1 | 1 | N/A |
| **Choice of health outcomes** | 1 | 1 | 1 | 1 | 1 | 1 | 1 | 1 |
| **Measurement of effectiveness** | 1 | 1 | 1 | 1 | 1 | 1 | 1 | 1 |
| **Measurement of preference-based outcomes** | 1 | 1 | 1 | 1 | N/A | ½ | 1 | 1 |
| **Resources and costs** | 1 | ½ | 1 | 1 | 1 | ½ | 1 | 1 |
| **Currency** | 1 | 1 | 1 | 1 | 1 | ½ | 1 | 1 |
| **Choice of model** | 1 | N/A | N/A | N/A | N/A | N/A | N/A | ½ |
| **Assumptions** | 1 | N/A | N/A | N/A | N/A | N/A | N/A | 1 |
| **Analysis** | 1 | 1 | 1 | 1 | ½ | 1 | 1 | 1 |
| **Parameters** | 1 | ½ | ½ | ½ | ½ | ½ | 1 | 1 |
| **Incremental costs and outcomes** | 1 | 1 | 1 | 1 | 1 | 1 | 1 | 1 |
| **Uncertainty** | 1 | 1 | 1 | 1 | 0 | 1 | 1 | 1 |
| **Heterogeneity** | ½ | N/A | N/A | 1 | 0 | 1 | 1 | 1 |
| **Findings** | 1 | 1 | 1 | 1 | ½ | 1 | 1 | ½ |
| **Funding** | 1 | 0 | 1 | 1 | 0 | 0 | 1 | 1 |
| **Conflicts** | 1 | 1 | 1 | ½ | 1 | 1 | 1 | 1 |
| **Total** | **21/24** | **18/21** | **17/21** | **19/22** | **13.5/21** | **18/22** | **21/22** | **18/23** |
| **% score** | **88** | **86** | **81** | **86** | **64** | **82** | **95** | **78** |

0=not reported or insufficiently reported; ½ = reporting unclear or components not reported; 1 = sufficient reporting

**Table A4-4** CHEERS assessment of economic evaluations of pathogen identification interventions and other sepsis interventions

|  | **Pathogen identification** | | | | **Other** | |
| --- | --- | --- | --- | --- | --- | --- |
| **Author:** | **Lehmann 2010** | **Alvarez 2012** | **Stevenson 2016** | **Cambau 2017** | **Champunot 2014** | **Ward 2016** |
| **Title** | ½ | ½ | 1 | 1 | 1 | ½ |
| **Abstract** | ½ | ½ | ½ | 0 | ½ | ½ |
| **Background** | 1 | 1 | 1 | 1 | 1 | 1 |
| **Target population** | 0 | 1 | 1 | 1 | 0 | 1 |
| **Setting and location** | ½ | 1 | ½ | ½ | 1 | 1 |
| **Perspective** | 1 | 1 | 1 | 1 | 1 | ½ |
| **Comparators** | ½ | 1 | 1 | 1 | 1 | 1 |
| **Time horizon** | 0 | ½ | 1 | 1 | 0 | 0 |
| **Discounting** | 0 |  | 1 |  |  | 1 |
| **Choice of health outcomes** | 1 | 1 | 1 | 1 | ½ | 1 |
| **Measurement of effectiveness** | ½ | ½ | 1 | 1 | 1 | 1 |
| **Measurement of preference-based outcomes** | ½ | N/A | 1 | N/A | N/A | ½ |
| **Resources and costs** | 1 | 1 | 1 | ½ | ½ | 1 |
| **Currency** | ½ | 0 | ½ | 1 | 1 | 0 |
| **Choice of model** | ½ | N/A | 1 | N/A | 1 | 1 |
| **Assumptions** | ½ | N/A | 1 | N/A | 0 | ½ |
| **Analysis** | ½ | 1 | 1 | ½ | ½ | 1 |
| **Parameters** | ½ | 1 | 1 | ½ | 1 | 1 |
| **Incremental costs and outcomes** | ½ | 1 | ½ | ½ | 1 | 1 |
| **Uncertainty** | 1 | 1 | 1 | 1 | 1 | 1 |
| **Heterogeneity** | ½ | 0 | N/A | N/A | 0 | N/A |
| **Findings** | 1 | 1 | 1 | 0 | ½ | ½ |
| **Funding** | 1 | 0 | 1 | 1 | 0 | 1 |
| **Conflicts** | 1 | 0 | 1 | 1 | 1 | 1 |
| **Total** | **8/24** | **12/20** | **21/23** | **12/19** | **12/22** | **15/23** |
| **% score** | **33** | **60** | **91** | **63** | **55** | **65** |

0=not reported or insufficiently reported; ½ = reporting unclear or components not reported; 1 = sufficient reporting

**CHEERS assessment of economic evaluations of therapies no longer in clinical practice**

**Table A4-5** CHEERS assessment of economic evaluations of monoclonal antibodies

| **Author:** | **Schulman 1991** | **Barriere 1992** | **Badia 1993** | **Chalfin 1993** | **Chang 1993** | **Linden 1995** | **Wang 1999** |
| --- | --- | --- | --- | --- | --- | --- | --- |
| **Title** | 1 | ½ | 1 | 1 | ½ | 1 | 0 |
| **Abstract** | ½5 | 0 | ½ | 1 | 0 | ½ | ½ |
| **Background** | 1 | ½ | 1 | 1 | 1 | 1 | 1 |
| **Target population** | ½ | 0 | 1 | 1 | 1 | 1 | 1 |
| **Setting and location** | ½ | 1 | 1 | 1 | 1 | 1 | 0 |
| **Perspective** | ½ | 1 | 0 | 1 | 0 | ½ | 1 |
| **Comparators** | ½ | 0 | ½ | 1 | ½ | 1 | ½ |
| **Time horizon** | ½ | 0 | ½ | ½ | 0 | 0 | 1 |
| **Discounting** | 1 | 0 | 1 | N/A | N/A | 0 | N/A |
| **Choice of health outcomes** | 1 | 0 | 1 | 1 | ½ | 1 | 1 |
| **Measurement of effectiveness** | ½ | 0 | 1 | ½ | ½ | 1 | 1 |
| **Measurement of preference-based outcomes** | N/A | N/A | N/A | N/A | N/A | N/A | N/A |
| **Resources and costs** | 1 | 0 | 1 | ½ | ½ | 1 | 1 |
| **Currency** | 0 | ½ | 1 | 0 | 0 | 0 | ½ |
| **Choice of model** | ½ | 0 | 1 | 1 | N/A | ½ | 1 |
| **Assumptions** | 1 | 0 | 1 | 1 | N/A | 1 | 1 |
| **Analysis** | ½ | 0 | 1 | 1 | ½ | ½ | 1 |
| **Parameters** | 1 | ½ | 1 | 1 | 1 | 1 | 1 |
| **Incremental costs and outcomes** | ½ | ½ | ½ | 1 | 1 | ½ | ½ |
| **Uncertainty** | 1 | 0 | 1 | 1 | 0 | 1 | 1 |
| **Heterogeneity** | N/A | 1 | 1 | 0 | 1 | 1 | 1 |
| **Findings** | 1 | ½ | 1 | 1 | ½ | 1 | ½ |
| **Funding** | 0 | 0 | 1 | 1 | 0 | 0 | 0 |
| **Conflicts** | 0 | 0 | 0 | 1 | 0 | 0 | 0 |
| **Total** | **14/22** | **3/23** | **17/23** | **17/22** | **6/20** | **15.5/23** | **13/22** |
| **% score** | **64** | **13** | **74** | **77** | **30** | **67** | **59** |

0=not reported or insufficiently reported; ½ = reporting unclear or components not reported; 1 = sufficient reporting

**CHEERS assessment of economic evaluations of therapies no longer in clinical practice**

**Table A4-6** CHEERS assessment of economic evaluations of drotrecogin alfa (activated)

|  | **Manns 2002** | **Angus 2003** | **Betancourt 2003** | **Fowler 2003** | **Neilson 2003** | **Sacristan 2004** | **Davies 2005** | **Hjelmgren 2005** | **Franca 2006** | **Green 2006** | **Costa 2007** | **Dhainaut 2007** | **Sadique 2011** |
| --- | --- | --- | --- | --- | --- | --- | --- | --- | --- | --- | --- | --- | --- |
| **Title** | 1 | 1 | ½ | 1 | 1 | 1 | 1 | 1 | 1 | 1 | 1 | ½ | 1 |
| **Abstract** | ½ | 1 | 1 | ½ | 1 | 1 | 1 | ½ | ½ | ½ | 0 | ½ | ½ |
| **Background** | 1 | 1 | 1 | 1 | 1 | 1 | 1 | 1 | 1 | 1 | 1 | 1 | 1 |
| **Target population** | 1 | 1 | 1 | 1 | 1 | 1 | 1 | 1 | 1 | 1 | 1 | 1 | 1 |
| **Setting and location** | 1 | ½ | 1 | ½ | 1 | ½ | 1 | 1 | ½ | 1 | 0 | 1 | 1 |
| **Perspective** | 1 | 1 | 1 | 1 | 1 | 1 | 1 | 0 | 0 | 1 | 1 | 1 | 0 |
| **Comparators** | 1 | 1 | 1 | ½ | 1 | 1 | 1 | 1 | 1 | ½ | 1 | ½ | ½ |
| **Time horizon** | 1 | 1 | 1 | 1 | ½ | ½ | ½ | 0 | 1 | ½ | 1 | ½ | 1 |
| **Discounting** | 1 | 1 | N/A | 1 | 1 | 1 | 1 | 1 | 1 | 1 | 1 | 1 | 1 |
| **Choice of health outcomes** | 1 | 1 | 1 | 1 | 1 | 1 | 1 | 1 | 1 | 1 | 1 | 1 | 1 |
| **Measurement of effectiveness** | 1 | 1 | 1 | 1 | 1 | 1 | 1 | 1 | 1 | 1 | 1 | 1 | 1 |
| **Measurement of preference-based outcomes** | 1 | 1 | N/A | 1 | N/A | N/A | ½ | 1 | 1 | 1 | ½ | 1 | 1 |
| **Resources and costs** | 1 | 1 | ½ | 1 | 1 | 1 | 1 | 1 | 1 | 1 | 1 | 1 | 1 |
| **Currency** | 1 | 1 | 1 | 1 | ½ | 1 | 1 | 1 | 1 | ½ | 1 | ½ | ½ |
| **Choice of model** | ½ | N/A | 1 | 1 | 1 | ½ | 0 | 1 | ½ | 1 | 1 | N/A | N/A |
| **Assumptions** | 1 | N/A | 1 | 1 | 1 | 1 | 1 | ½ | 1 | 1 | 1 | N/A | N/A |
| **Analysis** | 1 | 1 | 1 | 1 | 1 | ½ | 0 | 0 | ½ | 1 | 1 | 1 | 1 |
| **Parameters** | 1 | 1 | 0 | 1 | 1 | 1 | 1 | 1 | ½ | 1 | 1 | 1 | 1 |
| **Incremental costs and outcomes** | ½ | 1 | 1 | 1 | 1 | 1 | 1 | ½ | ½ | ½ | ½ | 1 | 1 |
| **Uncertainty** | 1 | 1 | ½ | 1 | 1 | 1 | 1 | 1 | 1 | 1 | 1 | 1 | 1 |
| **Heterogeneity** | 1 | 1 | 1 | 1 | 1 | 1 | 0 | 1 | 1 | 1 | 1 | 0 | 1 |
| **Findings** | ½ | 1 | 1 | 1 | 1 | 1 | 1 | 1 | 1 | 1 | 1 | 1 | 1 |
| **Funding** | 1 | 1 | 1 | 1 | 1 | 0 | 1 | 0 | ½ | 1 | 0 | 1 | 0 |
| **Conflicts** | 0 | 1 | 1 | 1 | 0 | 0 | 1 | 0 | 0 | 1 | 1 | 1 | 1 |
| **Total** | **19/24** | **21/22** | **18/22** | **21/24** | **20/23** | **17/23** | **19/24** | **16/24** | **15/24** | **19/24** | **19/24** | **16/22** | **17/22** |
| **% score** | **79** | **95** | **82** | **88** | **87** | **74** | **79** | **67** | **63** | **79** | **79** | **73** | **77** |

0=not reported or insufficiently reported; ½ = reporting unclear or components not reported; 1 = sufficient reporting
